# Fragment Libraries Designed to Be Functionally Diverse Recover Protein Binding Information More Efficiently Than Standard Structurally Diverse Libraries

**DOI:** 10.1021/acs.jmedchem.2c01004

**Published:** 2022-08-12

**Authors:** Anna Carbery, Rachael Skyner, Frank von Delft, Charlotte M. Deane

**Affiliations:** † Oxford Protein Informatics Group, Department of Statistics, University of Oxford, Oxford OX1 3LB, U.K.; ‡ Diamond Light Source, Harwell Science and Innovation Campus, Didcot OX11 0DE, U.K.; § Centre for Medicines Discovery, University of Oxford, Oxford OX3 7DQ, U.K.

## Abstract

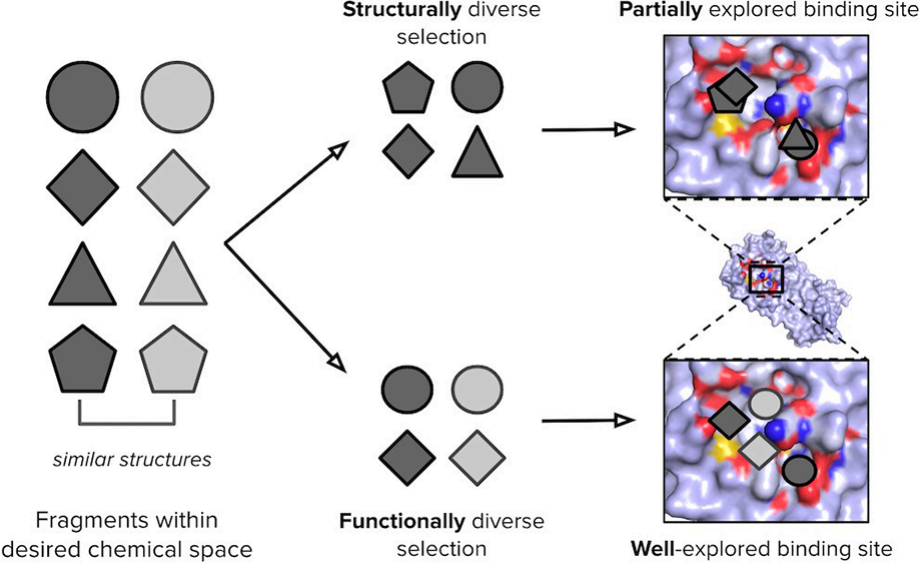

Current fragment-based drug design relies on the efficient exploration of chemical space by using structurally diverse libraries of small fragments. However, structurally dissimilar compounds can exploit the same interactions and thus be functionally similar. Using three-dimensional structures of many fragments bound to multiple targets, we examined if a better strategy for selecting fragments for screening libraries exists. We show that structurally diverse fragments can be described as functionally redundant, often making the same interactions. Ranking fragments by the number of novel interactions they made, we show that functionally diverse selections of fragments substantially increase the amount of information recovered for unseen targets compared to the amounts recovered by other methods of selection. Using these results, we design small functionally efficient libraries that can give significantly more information about new protein targets than similarly sized structurally diverse libraries. By covering more functional space, we can generate more diverse sets of drug leads.

## Introduction

Fragment-based drug design (FBDD) is now well-established as a powerful approach to early stage drug discovery and has led to success for targets that proved to be otherwise intractable.^1^ The first stage entails screening, in which libraries of fragments, compounds around a third of the size of typical drug-like molecules, are screened for binding to a protein of interest. The concept is that the small size of the fragments allows a more efficient search of chemical space and recovers more protein binding information than in traditional high-throughput screens, allowing the size of the library to be much smaller. This substantially reduces the number of experiments that need to be conducted within a screen.

Ideally, a fragment screen obtains information about the molecules or functional groups that bind the protein of interest and the interactions they make.^2^ Fragments are subsequently elaborated or combined to create larger lead molecules that can be developed into potential drugs. The better the site of interest is explored by fragments, the more insights we have into the key interactions critical for binding. As these key interactions are usually conserved upon generation of larger lead molecules,^3^ this translates to improved chances of a viable drug candidate being discovered.

### Design of Fragment Libraries

Maximizing the useful information that can be extracted from each fragment screening experiment is key, so design of fragment libraries is a major element of FBDD research.^4−8^ There are two major aspects to library design: definition of the desired region of chemical space and the sampling of that region of chemical space. Table S1 describes the design strategies and size of several major fragment libraries.

#### Definition of Desired Chemical Space

Fragment libraries tend to be built from molecules that adhere to the “rule of three”:^5^ fragments that have a molecular weight of <300 Da, fewer than three hydrogen-bond donors and acceptors, fewer than three rotatable bonds, and a cLogP of ≤3. Heavy atom counts tend to be limited to <20.^2^ These rules aim to limit the structural complexity of the fragments so that only one or two efficient interactions with the protein target are required for binding. Compounds containing toxicophores or highly reactive groups are not included,^9^ as these would not be appropriate fragments to use in the development of drug leads. Fragment libraries also tend to prioritize chemical tractability^10^ and/or the availability of analogues^11^ to enable fast and easy follow-up experiments. Such fragments that are ideal for subsequent lead development have recently been termed “social fragments”^12^ and are contrasted with “unsocial fragments” that have limited or no synthetic pathways for elaboration, and no analogues.

Historical experimental results can be incorporated to further guide their definition of desired chemical space. The SpotXplorer library^13^ was designed to contain pharmacophores that have been observed to commonly bind protein hot spots,^14^ based on a comprehensive analysis of the Protein Data Bank.^15^ A library designed recently^16^ used past experimental results to develop a machine learning model that generated novel fragments that contain the characteristics of fragments that bind multiple targets, termed “privileged fragments”.

Libraries may also have a particular intent, for example, to target certain protein classes or to fulfill specific properties. Examples of such properties include high Fsp^3^ character,^17^ a three-dimensional (3D) shape,^18^ the ability to form covalent bonds with the target,^19^ protein–protein interface binding character,^20^ or natural product resemblance.^17^

#### Sampling of Desired Chemical Space

To avoid the synthetic challenges presented by the design of novel compounds, most libraries are made using previously available fragments, whether available commercially or in house. In these cases, a catalogue of fragments that lie within the desired chemical space is generated, and fragments are selected from this in a way that maximizes the structural or shape diversity of the library. A common approach is to use molecular fingerprints such as ECFP,^21^ MACCS,^22^ and USRCAT.^23^ A fingerprint is generated for each fragment, and a maximin-derived algorithm^24^ (such as the RDKit MaxMin picker) is used to select the most structure or shape diverse fragments. For example, the DSiP library^25^ (the successor to ref (10)) uses USRCAT fingerprints while the F2X libraries^11^ use MACCS fingerprints to maximize structural and shape diversity.

Another method used to achieve structural diversity is to cluster fragments on the basis of structure or functional groups. Representatives of each cluster can then be selected for the final library. An advantage of this approach is that clusters that cover more attractive chemical space can be sampled more often than those that are less desirable. The 3D shape diverse library^26^ is an example of a library employing this strategy.

A few libraries consist of novel fragments that were designed and synthesized specifically for the library.^27,7^ The most common aim of such libraries is to address the historic uneven coverage of chemical space. Final compounds for the library are generally selected to ensure synthetic feasibility and a low degree of similarity to commercially available fragments and to maximize the shape diversity of the final library.^28^

In nearly all cases, chemists are reported to be the final gatekeeper of selection, using visual inspection.^16,29,30^ This indicates that algorithmic approaches are never trusted to completely select the fragments and makes it difficult to learn from these final decisions, which are rarely fully documented or quantified.

#### Influence of HTS Library Design on Fragment Library Design

To assess the effectiveness of current library design methods, it is useful to first understand how these strategies originated. The emphasis on structural diversity on library design appears to have its origin in the design of HTS libraries,^31^ where the large chemical space made computable metrics imperative in the selection of compounds. Experimental approaches like diversity-oriented synthesis^32^ also built up on this principle. The premises were transferred to fragment library design, but to the best of our knowledge, the underlying assumptions were never rigorously interrogated.

Gordon et al.^31^ proposed that HTS screening libraries should be iteratively redesigned on the basis of the results of screening campaigns. This has been applied to several libraries to guide their definition of “attractive chemical space”; however, only knowledge of which fragments produced hits in past experiments was utilized, with 3D information from the protein–fragment structures being ignored. For example, an analysis of results from screens using the Astex 2012 fragment library^33^ showed a need to focus on fragments with 10–14 heavy atoms and ensure that larger fragments are not overly complex. The Vernalis library was analyzed using the results of 12 fragment screening campaigns,^29^ and it was found that compounds with slightly lower molecular weights had higher hit rates. AstraZeneca observed a high rate of project failure between 2002 and 2008^34^ and used the results to iteratively improve the library in several ways: fragments that were prone to decomposition, highly reactive, or deemed “unattractive” for follow-up chemistry by medicinal chemists were all removed, while pharmacophoric and structural diversity analyses were employed to “fill in” gaps in chemical space.

### Functional Activity of Fragment Libraries

Using only binary hit or miss results does not tell us whether these frequently hitting fragments are giving us diverse information about a target, so this may fail in achieving the primary aim of a fragment screen of thoroughly exploring the binding site of a protein of interest. It is known that the molecular structure of a fragment does not accurately predict the interactions that it can make with a protein.^35^ Similar fragments may have diverse functional activity (e.g., they bind to different protein environments), while structurally diverse fragments may have very similar activity.^36^ Thus, the number of hits cannot be used as a proxy for the quantity of information.

Most library designs aim to elucidate as much information as possible about a protein’s binding site(s). However, so far it has been difficult to establish which or indeed whether any fragment libraries achieve this. Now that crystallographic fragment screens are routine, we can use primary data of protein–fragment interactions to assess whether structurally diverse libraries are behaving in a functionally diverse manner.

To establish whether fragment libraries designed to be structurally diverse have functional redundancy, we examined a set of 10 diverse targets that have all been screened against the majority of fragments in the DSiP library by XChem.^37^ The same set of fragments has been tested on the same targets, generating full data of what bound and how, as well as which fragments did not bind. Using these data, we describe an approach for analyzing functional redundancy within a fragment library and examine the relationship between structurally diverse and functionally diverse fragment libraries. Our findings suggest that structurally diverse fragment libraries do not necessarily exhibit any more functional diversity than randomly selected libraries. On the contrary, by selecting functionally diverse fragment libraries, we show that the information recovered for unseen targets is substantially improved compared with that obtained by using randomly selected or structurally diverse fragments.

## Results

Structural data from fragment screens of 10 unrelated protein targets bound to 520 fragments were used in this study. Protein–ligand interaction fingerprints (IFPs) were calculated for each structure, between fragment atoms and protein residues (residue IFP) and between fragment atoms and protein atoms (atomic IFP). For both types of IFP, the fragments were ranked on the basis of the novel interactions they formed with all or a subsection of protein targets (see Experimental Section). These rankings were used to group fragments for analysis, defined in Table 1.

**Table 1:** Definitions for the Various Groups of Fragments Used in This Analysis

group of fragments	definition
top 100	the 100 most informative fragments, as determined using the fragment ranking methods; also can be described as the most functionally diverse selection of fragments
remaining minimum	fragments not in the top 100 that still form novel interactions and are thus within the minimum number of fragments to form all interactions from the original screen
remaining bound	fragments not in the top 100 that have bound one or more protein targets
redundant	fragments that have bound to one or more protein targets yet do not form any novel interactions
never bound	fragments that have never been observed to bind a protein target
structurally diverse	sets of fragments that have been selected to be functionally diverse using MACCS similarity
random	sets of randomly selected fragments

We also examined the types and frequencies of different protein–ligand interactions made (shown in Figures S2–S4).

### Structurally Diverse Fragments Can Form Overlapping Interactions

**Figure 1:**
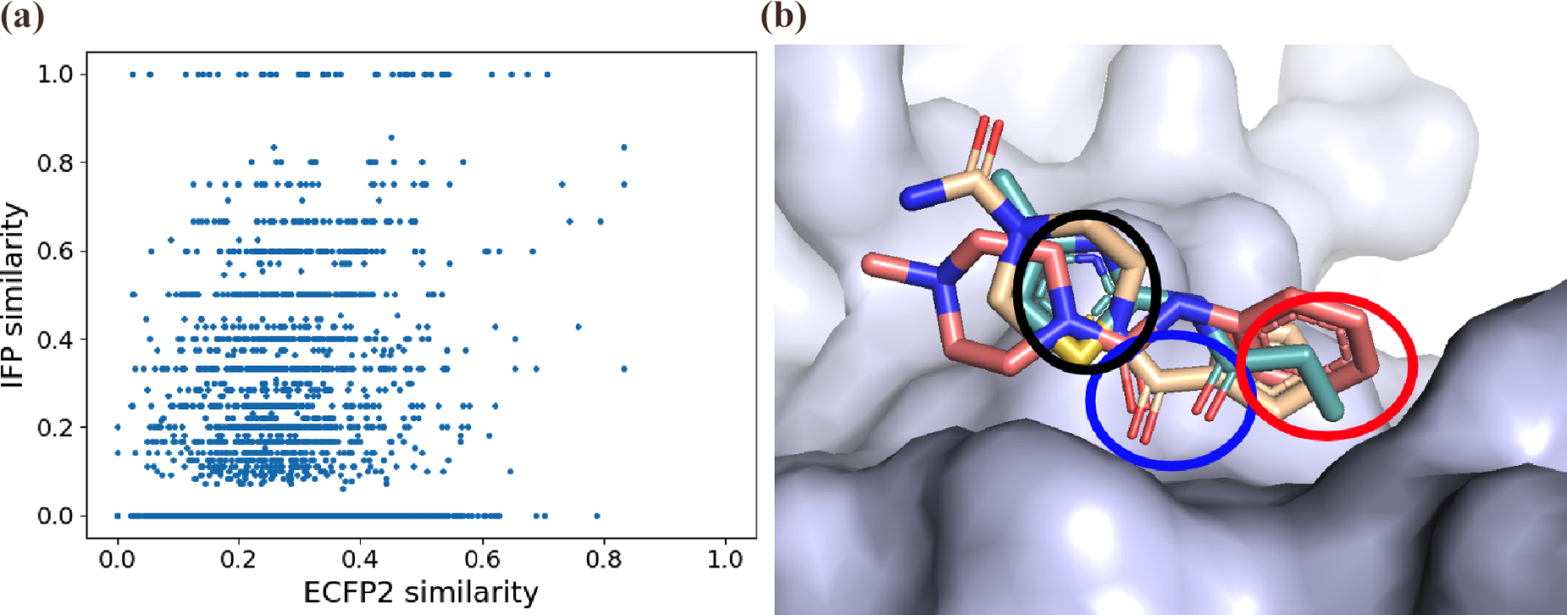
Very diverse fragments form identical interactions. (a) Molecular (ECFP2) similarity compared to functional (IFP) similarity. Each point represents a pair of fragments that bind to the same target. There is no direct correlation between ECFP2 (molecular) similarity and IFP (functional) similarity, showing that structural diversity of fragments is not predictive of functional diversity. (b) Example of three fragments that form the same interactions with target TBXTA (lilac). The atoms circled in bright blue (carbonyl oxygens) are forming hydrogen bonds with two residues. Atoms circled in black (nitrogens) are forming a hydrogen bond with a single residue. Atom groups circled in red are forming a hydrophobic interaction with a single residue.

We compared the molecular similarity (calculated using ECFP2 fingerprints^21^) to the residue IFP (our measure of functional activity) similarity for fragments bound to 10 highly diverse protein targets (Table S3). Fragments that are structurally very different (ECFP2 similarities as low as 0.02) bound to the same location on a target and form one or more of the same interactions, leading to an IFP similarity above zero (Figure 1a). Across the set, we found 44 pairs of structurally distinct fragments that formed identical interactions. An example of this structural dissimilarity but functional similarity is shown in Figure 1b, where three distinct fragments form identical interactions with the protein. The molecular fingerprint (ECFP2) similarity of these three fragments ranges between 0.27 and 0.34, which would be considered appropriately diverse for inclusion in conventional libraries.

While the 44 pairs of fragments that form identical interactions account for only 0.82% of all fragment pairs, more than one-fourth (26.9%) of fragment pairs shared at least one common interaction. To explore the possibility that fewer fragments could form the same interactions with the targets, we assessed which fragments formed the most novel interactions and calculated the minimum number of fragments required to explore all interactions across all targets.

### Ranking of Fragments Reveals Redundancy in Interactions

**Figure 2:**
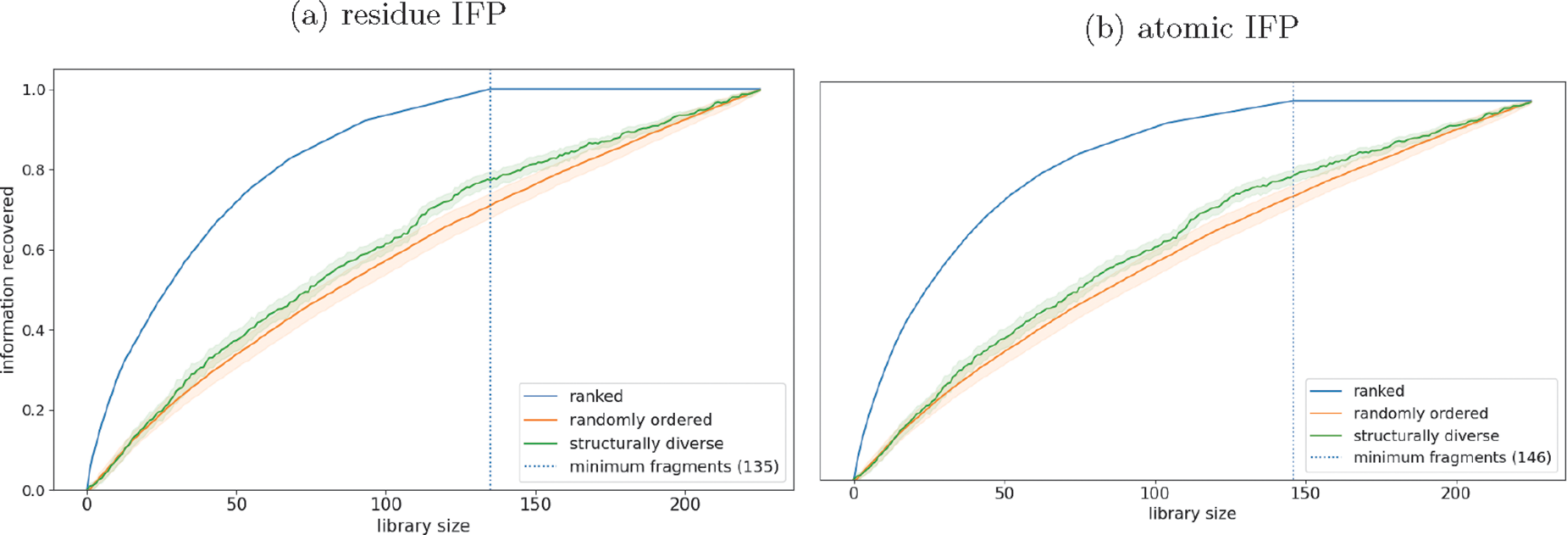
Ranked fragment libraries recover information at smaller library sizes than other methods. The blue dotted line indicates the minimum number of fragments required to recover all information. (a) Fragments have been ranked using the residue IFP method. (b) Fragments have been ranked using the atomic IFP method.

Fragments were ranked by the number of novel interactions (residue level and atomic level) that they formed with the 10 targets in the data set (Table S3). Two libraries, one randomly ordered and another structurally diverse, were generated for comparison to the functionally diverse libraries (see Experimental Section for details of how these were prepared). Each of these three methods of ordering fragments was repeated 100 times, and the mean fraction of unique interactions (also termed “information”) recovered at each library size across all runs was calculated (Figure 2).

Interactions were recovered at smaller library sizes for the functionally ranked library compared with either the random or the structurally diverse library. On a residue level, all interactions with targets were recovered using only the 135 top-ranked fragments of the 225 that were bound to at least one target (Figure 2a); for the atomic level of interactions, 146 fragments were required (Figure 2b). This result shows that upon selection of the fragments in this way, a 135-fragment screen could recover the same information as randomly selected 520-fragment screens on all 10 targets. It was expected that the ranked libraries (using both residue and atomic IFP) would recover information at smaller library sizes due to the method of ranking; however, it is notable that structurally diverse libraries do not recover information at library sizes any smaller than the randomly selected libraries. The lack of difference between structurally diverse and random libraries may be because the fragments used are already relatively structurally diverse.

To assess whether different libraries were selecting similar fragments, the mean number of fragments in common between libraries of 100 fragments was calculated. The functionally diverse and structurally diverse libraries had 49 fragments in common, similar to the 44 fragments in common between the functionally diverse and randomly selected fragments. This indicates that the level of similarity between the functionally diverse and structurally diverse libraries is little more than random. Conversely, the functionally diverse libraries generated using the residue IFP and atomic IFP methods have 83 fragments in common.

### Functionally Diverse Compounds Exhibit Chemical Properties Different from Those of Nonbinding Fragments

**Figure 3:**
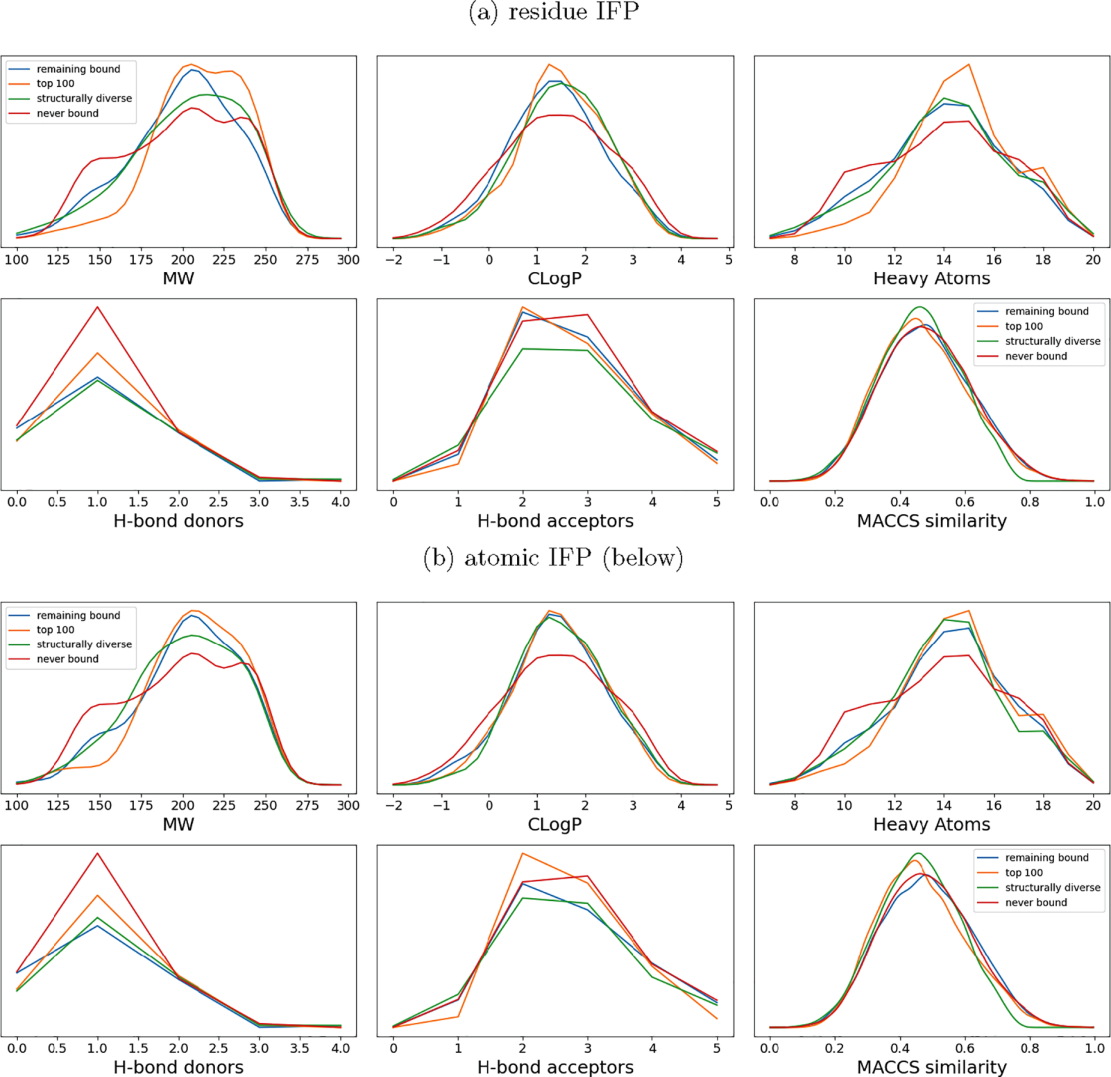
Fragments that have never bound to a target (red) are more likely to have very low or very high molecular weights and heavy atom counts. Fragments have been categorized into the “top 100” and “remaining bound” groups on the basis of our functional ranking (see Experimental Section). Fragments that bound no targets make up the “never bound” set, while a set of structurally diverse fragments are shown for comparison. Various properties of these sets of fragments are compared. (a) Fragments have been ranked using the residue IFP method. (b) Fragments have been ranked using the atomic IFP method. In panels a and b, medium-sized fragments are most likely to be highly informative.

To explore the relationship between particular chemical properties and a fragment’s rank, we split the DSiP fragment library into three sets: the 100 top-ranked fragments (those that are most informative), the 125 remaining bound fragments (fragments that had bound to one or more proteins), and the 295 fragments that had never bound to a target (Figure 3). This was performed on sets generated by both residue and atomic IFP methods, and as a comparison, chemical properties were also calculated for a structurally diverse set of 100 fragments.

Fragments ranked in the top 100 functionally diverse were more likely to have a molecular weight (MW) of ∼200 for both residue and atomic IFP methods, while the “other bound” groups of fragments were slightly more likely to have a MW of <175. For all other properties, there was no substantial difference between groups of fragments that had bound one or more targets. Fragments that had not been observed to bind any target were much more likely to have a low MW (<175) and a higher heavy atom count (≥17) compared to those that had bound one or more targets. The low MW of many “never bound” fragments suggests that they may be too small for reliable detection and modeling in many experiments. As expected, the fragments selected as a diverse subset had lower MACCS similarity with each other compared with the overall library and the highly ranked fragments.

### Promiscuous Binders Are Not Necessarily the Most Informative Fragments

**Figure 4:**
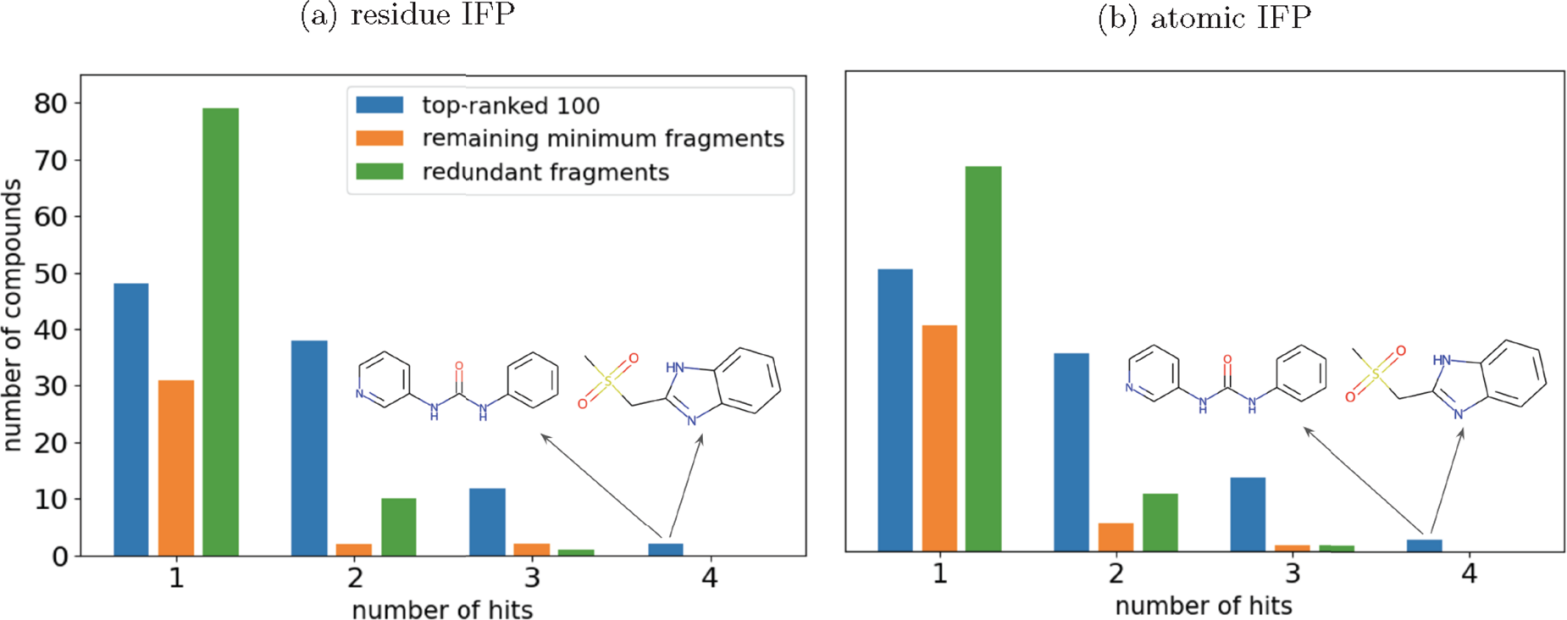
Number of targets bound by fragments in the DSiP library, grouped by their position when ranked. The 100 top-ranked fragments are compared to those remaining fragments that give us novel interactions and to fragments that form only redundant interactions. Promiscuous fragments (e.g., three or four hits) are not always selected as the most informative, whereas some fragments with only one hit are ranked in the top 100. Chemical structures of the two unique fragments that bound four targets are pictured within the figures. (a) Fragments have been ranked using the “residue IFP” method. (b) Fragments have been ranked using the “atomic IFP” method.

To examine whether a fragment that bound multiple targets (a promiscuous fragment) was likely to be selected as a highly informative fragment, we compared the number of hits (targets bound) for three sets of fragments: the 100 top-ranked fragments, the remaining minimum fragments (fragments that would be required to recover all information from the original screen), and the fragments that could be removed without losing any information (redundant fragments). While the top-ranked sets consisted mostly of fragments that had bound multiple targets, some fragments that bound to three or four targets were excluded in favor of those that had bound to only a single target (Figure 4).

### Functionally Diverse Fragments Recover Information More Efficiently from Unseen Targets

The results presented above show that a functionally diverse fragment set contains fragments different from those of a structurally diverse one. We investigated whether using such functionally diverse fragments is an effective strategy for more efficiently obtaining information about unseen targets. To do this, we performed a leave one out test (see Experimental Section for more details).

**Figure 5:**
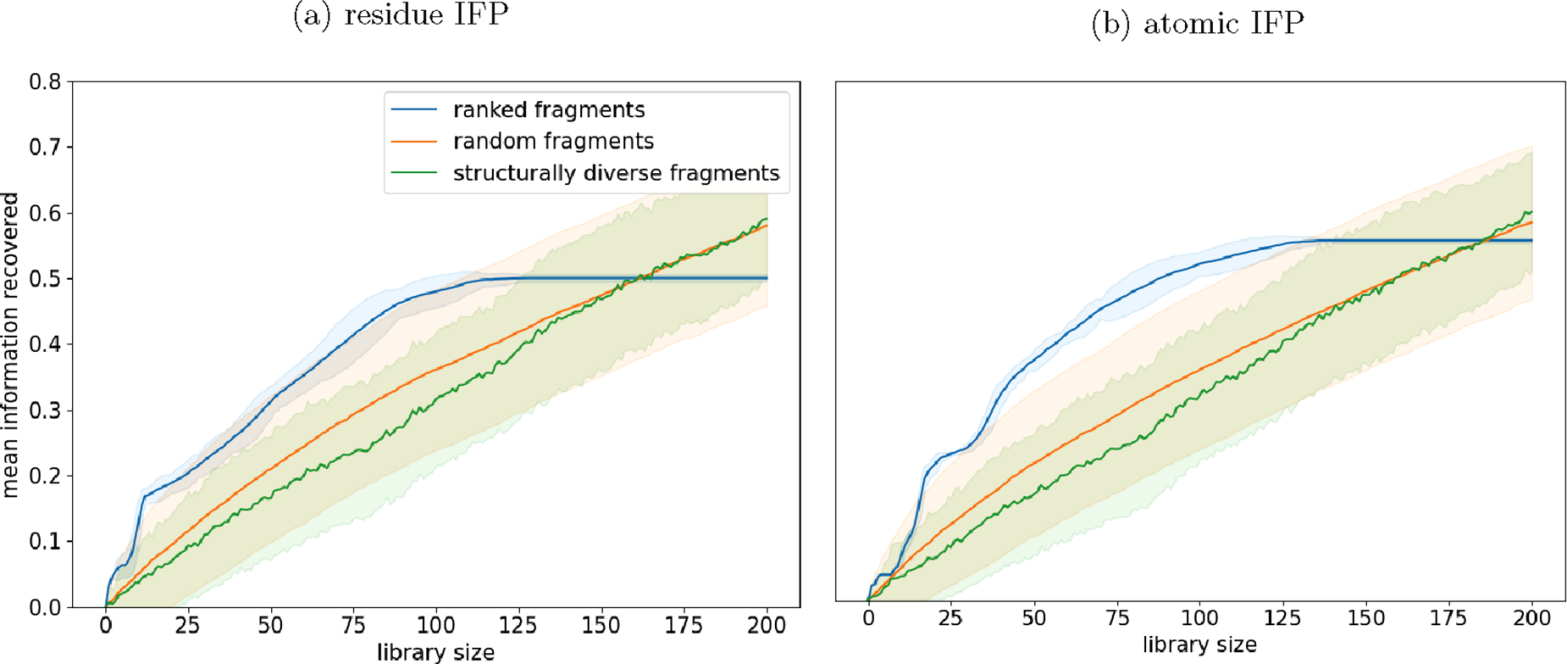
Ranked fragment libraries show superior information recovery for unseen targets at every library size. The recovery of information for each target when unseen was calculated 100 times. The mean across each run for each target was calculated, and the mean of these values was taken. This value is shown at each library size, with error clouds showing one standard deviation across 100 runs. (a) Fragments have been ranked using the “residue IFP” method. (b) Fragments have been ranked using the “atomic IFP” method.

**Figure 6:**
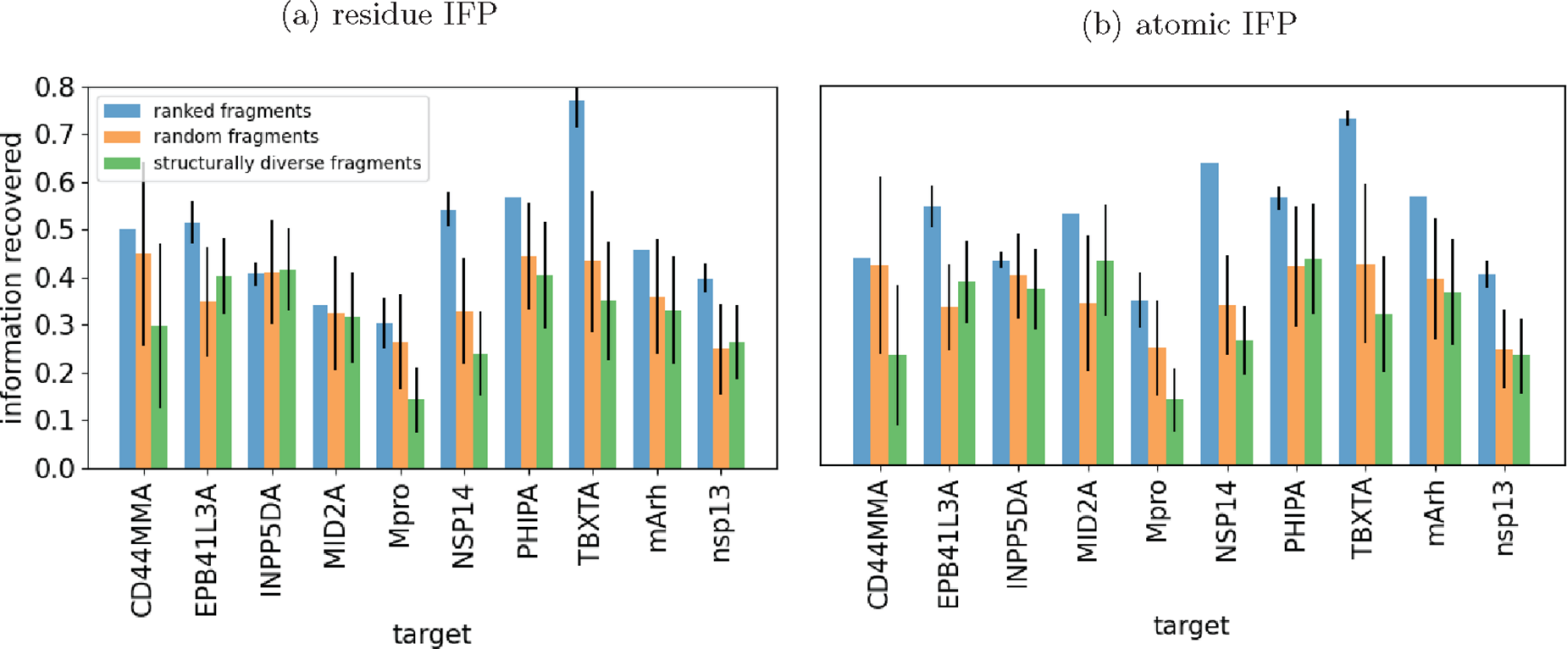
Top-ranked fragments show superior information recovery compared with random fragments and structurally diverse fragments. For each target, the average information recovery when using the 100 top-ranked fragments over 100 runs is shown. Error bars show one standard deviation. A lack of error bars shows no variability in the result. (a) Fragments have been ranked using the “residue IFP” method. (b) Fragments have been ranked using the “atomic IFP” method.

To compare the information recovery for each target when using functionally diverse fragments with the other methods of fragment selection, we analyzed the information recovered from each target at a library size of 100 fragments (Figure 6). We also calculated the fractional improvement when using the functionally diverse fragments compared with random and structurally diverse fragments (Figure S5). We compared the methods of fragment selection across all unseen targets. The mean values of information recovery across all targets are shown in Figure 5b. On average, the functional information about the unseen target was recovered more efficiently using the functionally diverse fragments than the random libraries.

### Targets Contribute Differently to the Prediction of Important Fragments for Unseen Targets

**Figure 7:**
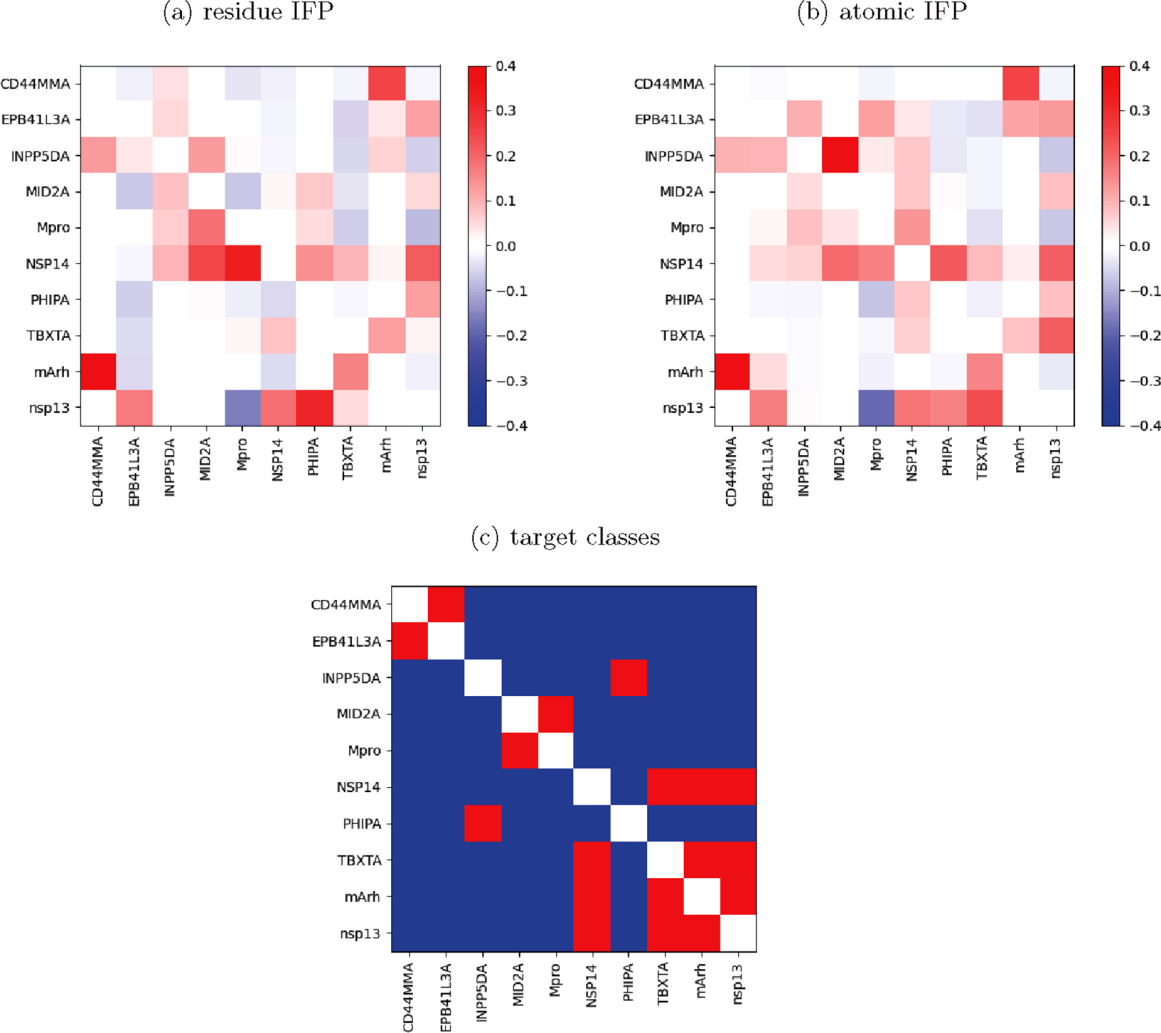
Impact of each target on every other target’s information recovery at library size of 100 fragments. For example, including mArh improves recovery of information about CD44MMA by 40% compared with only using the eight remaining targets to rank the fragments; including CD44MMA improves recovery of information about mArh by 27%. (a) Fragments have been ranked using the “residue IFP” method. (b) Fragments have been ranked using the “atomic IFP” method. (c) Heat map showing whether two targets fall into the same class. Red indicates that two targets are in the same class, and blue indicates a different class.

The targets in this data set are very diverse (the maximum pairwise global sequence identity is 27%), but some fragments do form similar interactions with different targets. To assess the impact of each target on the effectiveness of a fragment set for giving information about an unseen target, we removed the previously seen targets one by one, ranked only the nine remaining targets, and took the 100 top-ranked fragments as the functionally diverse library. The recovery of interactions was compared with the original (when results of all 10 targets were used to rank), and the factor that each target impacts the recovery of interactions for every other target was calculated. These impact scores are a measure of the similarity of the most informative fragments between two targets (Figure 7a,b). Between the residue IFP method and the atomic IFP method, the scores are consistent in terms of overall effect, but the magnitudes differ. Each target positively impacts some targets while negatively impacting others.

As a high impact score is indicative of similar fragments forming interactions with the targets, we assessed which targets were in the same classes (phosphatase/kinase, protease, nucleic, or other). This is shown in Figure 7c; however, it does not appear that there is a strong correlation between these two factors. This confirms that there are no two targets with similar fragment binding activity, yet by ranking fragments on previously seen targets, we can improve information recovery on unseen unrelated targets.

## Discussion

In agreement with previous work,^36^ we found that structurally diverse fragments can form similar or even identical interactions. Additionally, we have shown that by defining fragments as the interactions they make with all targets, we can select fragment libraries on the basis of their functional diversity. Such libraries canform more diverse interactions with previously unseen targets and thus improve the information recovered by an average of 68% and a maximum of 152% across all targets tested, compared with traditionally designed fragment libraries.

These findings suggest that selection based on structural diversity is not the optimal strategy when diverse functional information is desired. Given that suitable experimental data are now available for many fragments bound to multiple targets, it is feasible to explore new approaches to select fragments to screen previously unseen targets.

### Ranking Fragments Shows Redundancy in Interactions

To select the fragments that show the most diverse functional activity, we ranked fragments by the number of novel interactions they made with 10 targets. This showed that some fragments form far more novel interactions than others. We analyzed the most informative fragments (those that were most highly ranked) and compared them to less informative fragments and those that have never been seen to bind. Our analysis of the most informative fragments is broadly in agreement with previous work^38^ and suggests that fragments with molecular weights between 175 and 240 (and heavy atom counts between 12 and 16) perform optimally in fragment screens, perhaps as they can make multiple interactions without being structurally too complex, but are also large enough to be detected. Additionally, while promiscuous binders (fragments that bind multiple targets) are more likely to be considered as highly informative fragments, their promiscuity alone is not enough to guarantee this. This supports our hypothesis that using only information about which fragments have a high hit rate is not the most effective strategy for library redesign.

### Functionally Diverse Fragments Recover Information More Efficiently from Unseen Targets

We then proceeded to study the potential of the ranking protocol described above as a novel method for selecting fragments to screen on unseen targets. We tested whether a set of fragments that exhibited functional diversity in previously screened targets were more efficient in information recovery than a random set of fragments or a structurally diverse set of fragments on an unseen target. Both residue IFP and atomic IFP ranking methods achieved better information recovery than comparison methods at a library size of 100 fragments: the residue IFP method improved recovery by a maximum of 126% and an average of 59%, and the atomic IFP method improved recovery by a maximum of 153% and an average of 76%. The ranked library was less efficient at information recovery on only one target (INPP5DA), only when using the residue IFP method and by only 2%. At smaller library sizes, the average improvement in information recovery is even larger. This result indicates that the functional redundancy of the DSiP fragment library remains across diverse targets and that designing the library in a functionally diverse way can lead to more information being generated for a target from a screen compared with traditionally designed libraries.

The atomic IFP method gives slightly more consistent results than the residue IFP method. This suggests that the particular atom within a residue with which a fragment interacts is important, as the atomic IFP method captures this information whereas the residue IFP method does not.

### Different Targets Contribute Differently to Prediction of Important Fragments for Unseen Targets

Finally, we set out to understand whether particular targets contributed disproportionately to the performance of our method, thus indicating that there were targets with similarity in their fragment binding activity within the data set. By testing the impact of each target’s screening results on the recovery of information for every other target in a 100-fragment screen, we scored the similarity between the most informative fragments between each pair of targets. Each target positively impacts some targets while negatively impacting other. Some targets are mostly negatively impacted by others at a residue level while being unaffected or positively affected at an atomic level. As a measure of binding similarity, we compared this to substrate class of protein; however, there was little to no correlation. Considering the complexities of protein–ligand binding behavior, it is unlikely that a simple predictor of this behavior exists, so further research would be required to explore potential ways to predict such similarities between targets. Additionally, as we would expect, the impact of each target on each other target will change as more results are included and artifacts due to the relatively small data set used here are reduced.

## Conclusions

Currently, the most common strategy for fragment library design is to select the most structurally diverse set of fragments from those that lie in the desired chemical space, without considering structural results from previous fragment screens. In this study, we have shown that libraries designed on the basis of functional diversity recover information more efficiently from unseen targets than traditionally designed structurally diverse libraries.

Even with a limited data set, we have proven the potential for functionally diverse fragment selections to substantially improve the information recovered from fragment screens. Every time more experimental data become available, it would be possible to quickly reselect functionally diverse fragments that can reliably and significantly outperform traditional library selection methods.

This ability to better explore the interactions in protein binding sites would allow a larger number of diverse lead compounds to be developed, thus improving the chances of a fragment screening campaign producing a viable drug candidate. Additionally, we believe that by collecting comprehensive information about the functional activity of fragments (by testing them on many targets), we can begin to understand and predict which fragments will be functionally diverse just on the basis of their structure.

## Experimental Section

In this section, we describe how we rank fragments by their ability to give us the most information about key interactions and how we test the capacity of fragments we ranked highly to recover information about unseen targets.

### XChem Data Set

Structures of 309 protein–fragment complexes were used, from 10 targets and 225 unique fragments, representing results from 4928 individual crystals. Each target had between 13 and 65 fragment-bound structures. The targets were diverse, with a global pairwise sequence identity mean of 12% and a maximum of 27%. The sequence identity was calculated using EMBOSS Needle.^39^ No two targets shared a CATH class.^40^Table S3 contains descriptions of the targets used, and the fragment-bound structures of these are available on the Fragalysis platform.^41^ Full lists of fragments screened on the these targets are included within the Supporting Information.

To ensure that our analyses were not biased by fragments observed only a few times, we selected those fragments that had been tested on at least seven of our 10 targets. This resulted in 520 fragments being included, of which 295 had not bound to any targets. The remaining 225 fragments had bound to one to four targets. We selected fragments that had been tested on at least seven targets as a balance between coverage (seven of 10) and data set size. Requiring fragments to have been tested on eight or more of our targets would have led to the inclusion of only 480 fragments in the data set, of which only 214 had bound one or more targets. The fragments used in our analysis are included in the Supporting Information.

### Selection of Functionally Diverse Fragments

#### Definition of Functional Activity

We define the functional activity of a fragment as the interactions it forms with the protein. This definition is used to prioritize our understanding of a fragment’s ability to form interactions rather than the structure of the fragment itself. The interactions in each structure within the data set were calculated using ODDT’s InteractionFingerprint module,^42^ which generates a binary fingerprint for each protein–fragment structure. This method, in this study termed “residue IFP”, calculates up to eight types of interaction between the fragment and each residue in the protein (see Table S2 for details of interaction types). We also adapted the InteractionFingerprint module to output the interactions between the fragment and each atom of the protein, resulting in what we will term the “atomic IFP” method.

#### Ranking of Fragments Based on the Novelty of Functional Activity

The ranking protocol aims to identify fragments that add the most information about interactions a target can form. We start with a library size of one and append fragments one by one, each time including the fragment that adds the largest amount of novel information to the current library. As several fragments may add identical amounts of information, we repeat this 100 times, randomly shuffling the order of the fragment list before each run. At each library size, we calculate the number of interactions recovered compared to all of the interactions from the full screen. The mean fractional recovery rate at each library size is calculated, along with the standard deviation. The code used to rank fragments is available at https://github.com/oxpig/fragment-ranking.

### Other Methods of Fragment Ordering for Comparison

For comparison, we used two other methods of fragment ordering, including only fragments that had bound one or more targets, to match the set of fragments that were possible to rank. The first of these was a random control, where we shuffled the fragments into a random order, repeating this 100 times and calculating the mean and standard deviation of the recovery rate at each library size. We also generated a structurally diverse control, using the technique employed in the selection of the F2X-Entry library.^11^ We used RDKit^22^ to calculate the MACCS key for each fragment and MaxMin picker to select a structurally diverse set of fragments for every library size tested. This was also repeated 100 times with the mean recovery rate and standard deviation taken. This strategy for fragment selection was compared with other fingerprinting methods (shown in Figure S1).

### Testing a Fragment Ranking Protocol on Unseen Targets

To test the effectiveness of ranking fragments by their functional information, we tested the protocol on each target in the data set, using a leave one out test.

For each target, we ignored the results of its own screen, ranked the fragments using the other targets, and calculated the recovery of information about this previously ignored target. This was run 100 times, and the mean amount of information recovered was calculated, along with the standard deviation. These values were also calculated for randomly ordered and structurally diverse fragment libraries.

Only fragments that had bound to previously seen targets could be ranked, so once this library size was exceeded, “dummy fragments” were used, which did not recover any new information from the protein. This resulted in 100 sets of fragment libraries at every size from one to 200 for each of the library types. For each method, the mean information recovered was calculated, along with the standard deviation of this information recovery.

## Supporting Information Available

Hit rates for each fragment SMILES string in analysis (CSV)
All experiments (target–fragment SMILES) included in analysis (CSV)
Supplementary figures and tables (PDF)

## Abbreviations Used

FBDD: fragment-based drug design
ECFP: extended connectivity fingerprint
Fsp^3^: fraction of sp^3^ carbon atoms
IFP: interaction fingerprint
MACCS: Molecular ACCess System (keys)
USRCAT: ultrafast shape recognition with credo atom types

## References

[ref1] Erlanson D. A.; Fesik S. W.; Hubbard R. E.; Jahnke W.; Jhoti H. Twenty years on: the impact of fragments on drug discovery.” eng. Nat. Rev. Drug Discovery 2016, 15, 605–619. 10.1038/nrd.2016.109. 27417849

[ref2] Giordanetto F.; Jin C.; Willmore L.; Feher M.; Shaw D. E. Fragment hits: what do they look like and low do they bind?” eng. J. Med. Chem. 2019, 62, 3381–3394. 10.1021/acs.jmedchem.8b01855. 30875465 PMC6466478

[ref3] Chessari G.; Grainger R.; Holvey R. S.; Ludlow R. F.; Mortenson P. N.; Rees D. C. C-H functionalisation tolerant to polar groups could transform fragment-based drug discovery (FBDD). Chem. Sci. 2021, 12, 11976–11985. 10.1039/D1SC03563K. 34667563 PMC8457390

[ref4] Hann M M.; Leach A R.; Harper G. Molecular complexity and its impact on the probability of finding leads for drug discovery.” eng. J. Chem. Inf. Model. 2001, 41, 856–864. 10.1021/ci000403i. 11410068

[ref5] Congreve M.; Carr R.; Murray C.; Jhoti H. A ’rule of three’ for fragment-based lead discovery?” eng. Drug Discovery Today 2003, 8, 876–877. 10.1016/S1359-6446(03)02831-9. 14554012

[ref6] Keseru G. M.; Erlanson D. A.; Ferenczy G. G.; Hann M. M.; Murray C. W.; Pickett S. D. Design principles for fragment libraries: maximizing the value of learnings from pharma fragment-based drug discovery (FBDD) programs for use in academia. J. Med. Chem. 2016, 59, 8189–8206. 10.1021/acs.jmedchem.6b00197. 27124799

[ref7] Ray P. C.; Kiczun M.; Huggett M.; Lim A.; Prati F.; Gilbert I. H.; Wyatt P. G. Fragment library design, synthesis and expansion: nurturing a synthesis and training platform. Drug Discovery Today 2017, 22, 43–56. 10.1016/j.drudis.2016.10.005. 27793744

[ref8] Imbernon J. R.; Jacquemard C.; Bret G.; Marcou G.; Kellenberger E. Comprehensive analysis of commercial fragment libraries. RSC Med. Chem. 2022, 13, 300–310. 10.1039/D1MD00363A. 35434627 PMC8942207

[ref9] Konteatis Z. What makes a good fragment in fragment-based drug discovery?. Expert Opin. Drug Discovery 2021, 16, 723–726. 10.1080/17460441.2021.1905629. 33769176

[ref10] Cox O. B.; Krojer T.; Collins P.; Monteiro O.; Talon R.; Bradley A.; Fedorov O.; Amin J.; Marsden B. D.; Spencer J.; von Delft F.; Brennan P. E. A poised fragment library enables rapid synthetic expansion yielding the first reported inhibitors of PHIP(2), an atypical bromodomain. Chem. Sci. 2016, 7, 2322–2330. 10.1039/C5SC03115J. 29910922 PMC5977933

[ref11] Wollenhaupt J.; Metz A.; Barthel T.; Lima G. M. A.; Heine A.; Mueller U.; Klebe G.; Weiss M. S. F2X-Universal and F2X-Entry: structurally diverse compound libraries for crystallographic fragment screening. Structure 2020, 28, 694–706.e5. 10.1016/j.str.2020.04.019. 32413289

[ref12] St. Denis J. D.; Hall R. J.; Murray C. W.; Heightman T. D.; Rees D. C. Fragment-based drug discovery: opportunities for organic synthesis. RSC Med. Chem. 2021, 12, 321–329. 10.1039/d0md00375a. PMC8130625 34041484

[ref13] Bajusz D.; Wade W. S.; Satała G.; Bojarski A. J.; Ilaš J.; Ebner J.; Grebien F.; Papp H.; Jakab F.; Douangamath A.; Fearon D.; von Delft F.; Schuller M.; Ahel I.; Wakefield A.; Vajda S.; Gerencsér J.; Pallai P.; Keserű G. M. Exploring protein hotspots by optimized fragment pharmacophores. Nat. Commun. 2021, 12, 3201 10.1038/s41467-021-23443-y. 34045440 PMC8159961

[ref14] Ngan C. H.; Bohnuud T.; Mottarella S. E.; Beglov D.; Villar E. A.; Hall D. R.; Kozakov D.; Vajda S. FTMAP: extended protein mapping with user-selected probe molecules. Nucleic Acids Res. 2012, 40, W271–W275. 10.1093/nar/gks441. 22589414 PMC3394268

[ref15] Berman H. M.; Westbrook J.; Feng Z.; Gilliland G.; Bhat T N.; Weissig H.; Shindyalov I. N.; Bourne P. E. The protein data bank. Nucleic Acids Res. 2000, 28, 235–242. 10.1093/nar/28.1.235. 10592235 PMC102472

[ref16] Bilsland A. E.; McAulay K.; West R.; Pugliese A.; Bower J. Automated generation of novel fragments using screening data, a dual SMILES autoencoder, transfer learning and syntax correction. J. Chem. Inf. Model. 2021, 61, 2547–2559. 10.1021/acs.jcim.0c01226. 34029470

[ref17] Aimon A.; Karageorgis G.; Masters J.; Dow M.; Craven P. G. E.; Ohsten M.; Willaume A.; Morgentin R.; Ruiz-Llamas N.; Lemoine H.; Kalliokoski T.; Eatherton A. J.; Foley D. J.; Marsden S. P.; Nelson A. Realisation of small molecule libraries based on frameworks distantly related to natural products. Org. Biomol. Chem. 2018, 16, 3160–3167. 10.1039/C8OB00688A. 29645063

[ref18] Kidd S. L.; Fowler E.; Reinhardt T.; Compton T.; Mateu N.; Newman H.; Bellini D.; Talon R.; McLoughlin J.; Krojer T.; Aimon A.; Bradley A.; Fairhead M.; Brear P.; Díaz-Sáez L.; McAuley K.; Sore H. F.; Madin A.; O’Donovan D. H.; Huber K. V. M.; Hyvönen M.; von Delft F.; Dowson C. G.; Spring D. R. Demonstration of the utility of DOS-derived fragment libraries for rapid hit derivatisation in a multidirectional fashion. Chem. Sci. 2020, 11, 10792–10801. 10.1039/D0SC01232G. 34094333 PMC8162264

[ref19] Resnick E.; Bradley A.; Gan J.; Douangamath A.; Krojer T.; Sethi R.; Geurink P. P.; Aimon A.; Amitai G.; Bellini D.; Bennett J.; Fairhead M.; Fedorov O.; Gabizon R.; Gan J.; Guo J.; Plotnikov A.; Reznik N.; Ruda G. F.; Díaz-Sáez L.; Straub V. M.; Szommer T.; Velupillai S.; Zaidman D.; Zhang Y.; Coker A. R.; Dowson C. G.; Barr H. M.; Wang C.; Huber K. V. M.; Brennan P. E.; Ovaa H.; von Delft F.; London N. Rapid covalent-probe discovery by electrophile-fragment screening. J. Am. Chem. Soc. 2019, 141 (22), 8951–8968. 10.1021/jacs.9b02822. 31060360 PMC6556873

[ref20] Enamine. Enamine PPI fragment library. https://enamine.net/compound-libraries/fragment-libraries/ppi-fragments.

[ref21] Rogers D.; Hahn M. Extended-connectivity fingerprints. J. Chem. Inf. Model. 2010, 50, 742–754. 10.1021/ci100050t. 20426451

[ref22] Landrum G. RDKit: Open-source cheminformatics.

[ref23] Schreyer A. M.; Blundell T. USRCAT: real-time ultrafast shape recognition with pharmacophoric constraints. J. Cheminf. 2012, 48, 27 10.1186/1758-2946-4-27. PMC3505738 23131020

[ref24] Johnson M.; Lajiness M.; Maggiora G. Molecular similarity: a basis for designing drug screening programs. Prog. Clin. Biol. Res. 1989, 291, 167–171. 2726840

[ref25] XChem at Diamond Light Source. The Diamond-SGC iNext Poised library.

[ref26] Enamine. Enamine Fragment Collection. https://enamine.net/compound-libraries/fragment-libraries/.

[ref27] Hung A. W.; Ramek A.; Wang Y.; Kaya T.; Wilson J. A.; Clemons P. A.; Young D. W. Route to three-dimensional fragments using diversity-oriented synthesis. Proc. Natl. Acad. Sci. U. S. A. 2011, 108, 6799–6804. 10.1073/pnas.1015271108. 21482811 PMC3084099

[ref28] Downes T. D.; Jones S. P.; Klein H. F.; Wheldon M. C.; Atobe M.; Bond P. S.; Firth D.; Chan N. S.; Waddelove L.; Hubbard R. E.; Blakemore D. C.; De Fusco C.; Roughley S. D.; Lewis R.; Whatton M. A.; Woolford A. J.-A.; Wrigley G. L.; O'Brien P. Design and synthesis of 56 shape-diverse 3D fragments. Chem. - Eur. J. 2020, 26, 8969–8975. 10.1002/chem.202001123. 32315100 PMC7496344

[ref29] Chen I-J.; Hubbard R. E. Lessons for fragment library design: analysis of output from multiple screening campaigns. J. Comput.-Aided Mol. Des. 2009, 23, 603–620. 10.1007/s10822-009-9280-5. 19495994

[ref30] Lau W. F.; Withka J. M.; Hepworth D.; Magee T. V.; Du Y. J.; Bakken G. A.; Miller M. D.; Hendsch Z. S.; Thanabal V.; Kolodziej S. A.; Xing L.; Hu Q.; Narasimhan L. S.; Love R.; Charlton M. E.; Hughes S.; van Hoorn W. P.; Mills J. E. Design of a multi-purpose fragment screening library using molecular complexity and orthogonal diversity metrics. J. Comput.-Aided Mol. Des. 2011, 25, 621 10.1007/s10822-011-9434-0. 21604056

[ref31] Gordon E. M.; Kerwin J. F. Combinatorial chemistry and molecular diversity in drug discovery; Wiley-Liss: New York, 1998.

[ref32] Schreiber S. L. Molecular diversity by design. Nature 2009, 457 (7226), 153–154. 10.1038/457153a. 19129834

[ref33] Hall R. J.; Mortenson P. N.; Murray C. W. Efficient exploration of chemical space by fragment-based screening”. eng. Prog. Biophys. Mol. Biol. 2014, 116, 82–91. 10.1016/j.pbiomolbio.2014.09.007. 25268064

[ref34] Blomberg N.; Cosgrove D. A.; Kenny P. W.; Kolmodin K. Design of compound libraries for fragment screening. J. Comput.-Aided Mol. Des. 2009, 23, 513–525. 10.1007/s10822-009-9264-5. 19283339

[ref35] Guha R. On exploring structure-activity relationships. Methods Mol. Biol. 2013, 993, 81–94. 10.1007/978-1-62703-342-8_6. 23568465 PMC4852705

[ref36] Martin Y. C.; Kofron J. L.; Traphagen L. M. Do structurally similar molecules have similar biological activity?. J. Med. Chem. 2002, 45, 4350–4358. 10.1021/jm020155c. 12213076

[ref37] XChem at Diamond Light Source. XChem Fragment Screening. https://www.diamond.ac.uk/Instruments/Mx/Fragment-Screening.html.

[ref38] Kirsch P.; Hartman A. M.; Hirsch A. K. H.; Empting M. Concepts and core principles of fragment-based drug design. Molecules 2019, 24, 4309 10.3390/molecules24234309. PMC6930586 31779114

[ref39] Needleman S. B.; Wunsch C. D. A general method applicable to the search for similarities in the amino acid sequence of two proteins. J. Mol. Biol. 1970, 48, 443–453. 10.1016/0022-2836(70)90057-4. 5420325

[ref40] Sillitoe I.; Bordin N.; Dawson N.; Waman V. P; Ashford P.; Scholes H. M; Pang C. S M; Woodridge L.; Rauer C.; Sen N.; Abbasian M.; Le Cornu S.; Lam S. D.; Berka K.; Varekova I. H.; Svobodova R.; Lees J.; Orengo C. A CATH: increased structural coverage of functional space. Nucleic Acids Res. 2021, 49, D266–D273. 10.1093/nar/gkaa1079. 33237325 PMC7778904

[ref41] Skyner R.; von Delft F. Fragalysis. https://fragalysis.diamond.ac.uk/.

[ref42] Wójcikowski M.; Zielenkiewicz P.; Siedlecki P. Open drug discovery toolkit (ODDT): a new open-source player in the drug discovery field. J. Cheminf. 2015, 7, 26 10.1186/s13321-015-0078-2. PMC4475766 26101548

