# Supplementary material for: Fragment Libraries Designed to Be Functionally Diverse Recover Protein Binding Information More Efficiently Than Standard Structurally Diverse Libraries
Source: J Med Chem. 2022 Aug 12;65(16):11404–13. doi: 10.1021/acs.jmedchem.2c01004 (PMC9421645; doi:10.1021/acs.jmedchem.2c01004)
Supplement: Supplementary file 3 — jm2c01004_si_003.pdf [file jm2c01004_si_003.pdf]

Supporting Information

Fragment libraries designed to be functionally diverse recover protein binding information more efficiently than standard structurally diverse libraries

Anna Carbery[1, 2], Rachael Skyner[2], Frank von Delft[2, 3], and Charlotte M. Deane*[1]

[1]Oxford Protein Informatics Group, Department of Statistics, University of Oxford, Oxford OX1 3LB, UK
[2]Diamond Light Source, Harwell Science and Innovation Campus, Didcot OX11 0DE, UK
[3]Centre for Medicines Discovery, University of Oxford, Oxford OX3 7DQ, UK

* Corresponding author: deane@stats.ox.ac.uk

Contents

1 Supporting figures and tables **S1**

2 Molecules included in analysis **S6**

1 Supporting figures and tables

(a) residue IFP (b) atomic IFP

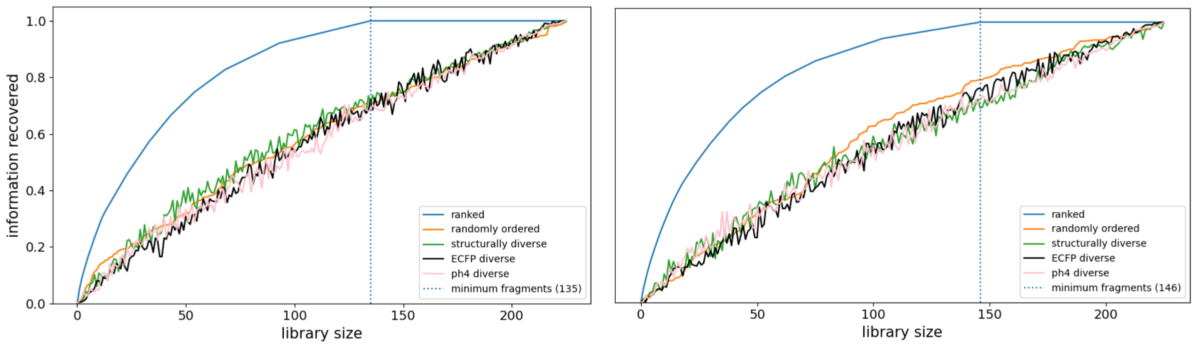

Figure S1: A comparison of the fragment ranking method (blue) with various types of structurally diverse fragment selection methods. MACCS keys (green), ECFP2 fingerprints (black) and 2D pharmacophoric fingerprints (pink) were generated for each fragment (all using RDKit [43]) and diverse selections of fragments based on these fingerprints were selected at each library size 5 times. The average information recovered from all targets at each library size for each type of fingerprint was calculated and this is shown. None of the structurally diverse fragment selection methods significantly outperformed the random control (orange). **(a):** fragments have been ranked using the residue IFP method. **(b):** fragments have been ranked using the atomic IFP method.

Library Name	Chemical space	Sampling strategies	Size	Reference
DSiP	poised	shape diversity (USRCAT)	768	[1]
F2X	pharmacophore	ph4 diversity (MACCS)	96	[2]
SpotXplorer	hotsplot exploration	ph4 + interaction clustering	96	[3]
Fraglites	halogenated fragments with paired H-binding motifs		25	[4]
PepLites	halogenated peptidomimetics		25	[5]
Cambridge 3D	3D and poised	diversity oriented synthesis	137	[6]
York 3D	3D, substituted aliphatic heterocycle	shape diversity	106	[7]
Leeds 3D	3D, natural product-like scaffolds, high sp3			[8][9]
MiniFrags	Astex's pharmacophores, ultra-low-MW	'chemical diversity'	81	[10]
Astex	general	chemical and structural diversity	2000	[11]
CovHetFrags	Small heterocyclic electrophiles	chemical properties	141	[12][13]
CysElectrophile	Cysteine covalent library		993	[14]
Novartis	general	structural diversity, clustering	1408	[15]
AZ	general xrc	structural diversity (ECFI)	384	[16]
Chembridge	large set	subsets: solubility, fluorine, bromine, structurally diverse	15000	[17]
Asinex	"BioDeisgn" - synthetically feasuble, NP-like and common NP ph4		20061	[18]
Life Chemicals	known bioactives	diversity, Fsp3, target family-specific	8000	[19]
Prestwick	Fragments of approved drugs		1456	[20]
Selcia			1214	[21]
Timetec		structural diversity		[22]
Zenobia	general inc. PPI	shape diverse	352	[23]
Bilsland	ML-generated privileged fragments	FeCo, novelty, 'examination by medicinal chemists'	741	[24]
Essential	initial screen, frequently reported hits		320	[25]
High fidelity	MedChem tractable	diversity selection using clustering algorithm	1920	[25]
Fluorinated	19F NMR		1000	[25]
Covalent	Diverse covalent		6210	[25]
Fully-functionalized	Photoaffinity labeled fragments		2000	[25]
NP-like	NP-like	scaffold frequency analysis	4160	[25]
3D shape-diverse	3D diverse	K-mean clustering, centroids taken	1200	[25]
PPI	peptidomimetics		3600	[25]
Single ph4	ro3-like	variety of ph4s and scaffolds	1500	[25]
Carboxylic acid	specific protein targets		4000	[25]
Halogen-enriched	halogen bonding motifs	diversity	3000	[25]
Fully-functionalized probe	Efficient exploration of novel protein targets w/ photoaffinity probes		640	[25][26]
EU openscreen	avilability of parent molecules within larger collection		1056	[27]
Maybridge	pharmacophorically-rich	clustering and centriods selected	2500	[28]
Vernalis	general	diversity of ph4 triangles	1063	[29]
Pfizer	general	diversity and visual inspection	2592	[30]

Table S1: Many of the fragment libraries currently in use. Some are used privately, while others are commercially available. Empty cells are indicative that the method has not been published.

Bit	Interaction type
0	hydrophobic contacts
1	aromatic face to face
2	aromatic edge to face
3	hydrogen bond (protein as hydrogen bond donor)
4	hydrogen bond (protein as hydrogen bond acceptor)
5	salt bridges (protein positively charged)
6	salt bridges (protein negatively charged)
7	salt bridges (ionic bond with metal ion)

Table S2: The eight types of protein-ligand interaction detected by ODDT's InteractionFingerprint module [31].

**Acronym**	**Full name**	**Description of activity**	CATH class(es)	**Reference**
**CD44MMA**	Hyaluronan binding domain of murine CD44	Binds hyaluronan via a lectin-like fold termed the Link module after appropriate functional activation	3.10.100.10	[32]
**EPB41L3A**	Erythrocyte membrane protein band 4.1-like 3a	Binds CD44 and transmits signals to the cytoskeleton	3.10.20.90, 1.20.80.10, 2.30.29.30	[33]
**INPP5DA**	Src homology 2 domain containing inositol polyphosphate 5-phosphatase 1 (SHIP1)	Phosphatase within the PI3K/AKT/mTOR signallling pathway	3.60.10.10	[34]
**MID2A**	Homo sapiens midline 2	Probable E3 ubiquitin-protein ligase	2.60.120.920	[35][36]
**Mpro**	SARS-CoV-2 Main Protease	Cysteine viral protease that cleaves at 11 sites, essential to viral replication	2.40.10.10, 1.10.1840.10	[5]
**NSP14**	SARS-CoV-2 nsp14 exoribonuclease	3-to-5 proofreading exoribonuclease (ExoN) and guanine-N7-methyltransferase	-	[37]
**PHIPA**	Pleckstrin Homology Domain Interacting Protein (second bromodomain)	Mediates transcriptional responses in pancreatic islet cells	1.20.920.10	[38]
**TBXTA**	T-box transcription factor TA	Transcription factor in embryonic tissues	2.60.40.820	[39]
**mArh**	SARS-CoV-2 non-structural protein 3	counteracts host-mediated antiviral adenosine diphosphate–ribosylation signaling	3.40.220.10	[40]
**nsp13**	SARS-CoV-2 non-structural protein 13	Superfamily 1 DNA or RNA helicase	3.40.50.300	[41]

Table S3: Descriptions of each target used in this study. CATH classes obtained through sequence-based searching of CATH [42] at https://www.cathdb.info/.

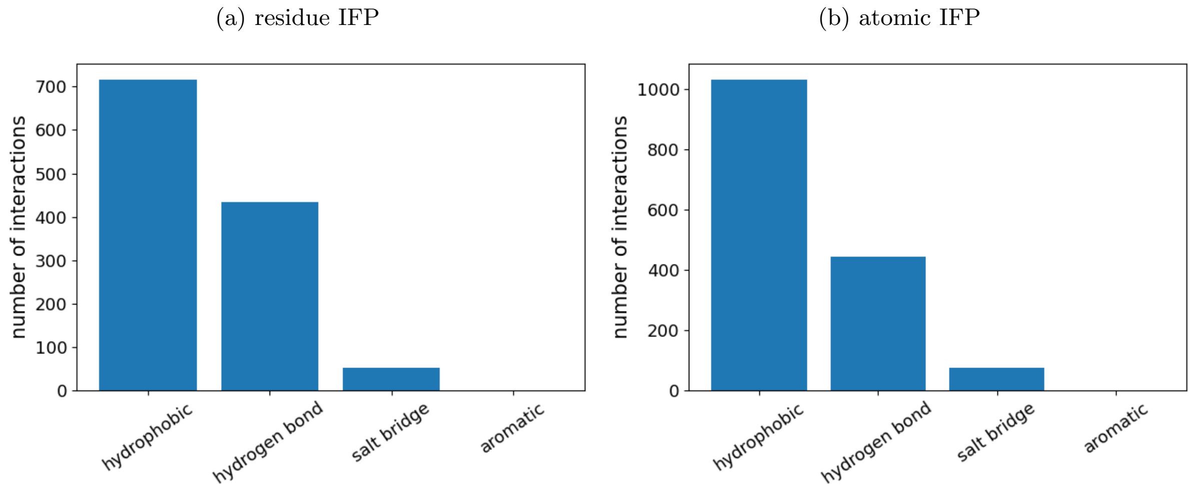

Figure S2: Interactions calculated across all protein-fragment structures used in this analysis. As in [44], most interactions are hydrophobic, followed by hydrogen bonds and salt bridges. No aromatic interactions were detected. **(a):** interactions were calculated using the residue IFP method. **(b):** fragments have been calculated using the atomic IFP method.

(a) residue IFP (b) atomic IFP

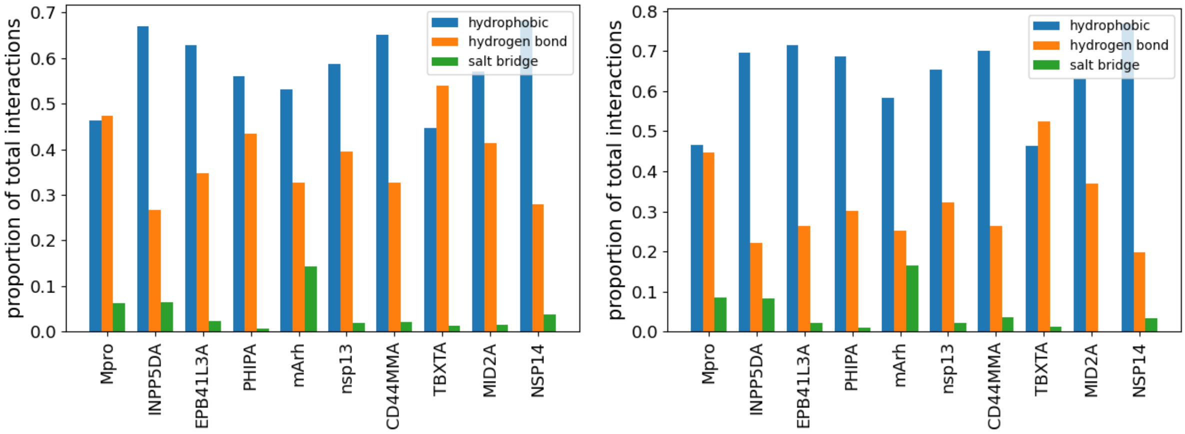

Figure S3: Interactions calculated for each target. Most targets follow the trend of making significantly more hydrophobic interactions than hydrogen bonds, however Mpro has similar levels of the two interaction types, and TBXTA forms more hydrogen bonds than hydrophobic interactions. **(a):** interactions were calculated using the residue IFP method. **(b):** fragments have been calculated using the atomic IFP method.

(a) residue IFP (b) atomic IFP

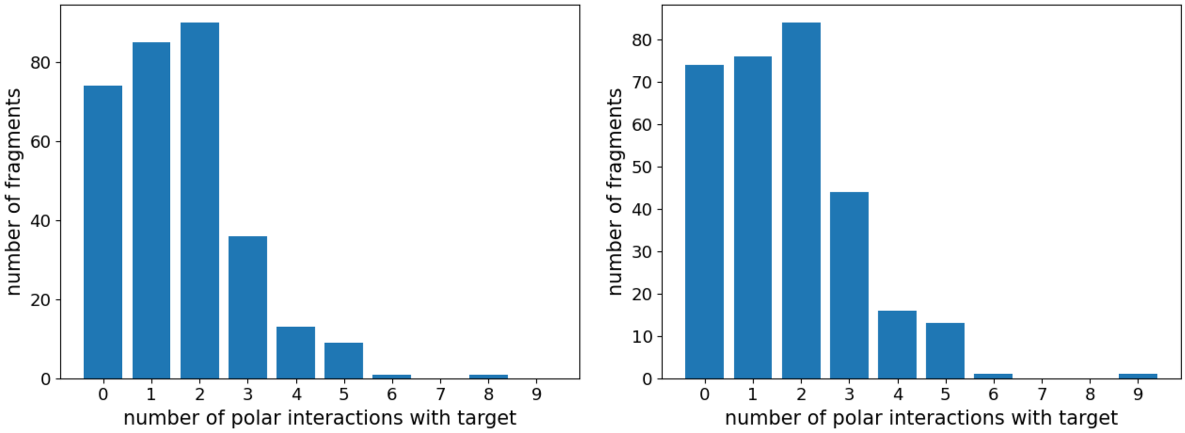

Figure S4: Number of polar interactions made by each fragment in a protein-fragment complex. In comparison with a previous analysis [45], this dataset exhibits a larger proportion of complexes in which no polar interactions are made with the protein. This could be due to the fact that water-mediated interactions were excluded from this analysis, or that a larger proportion of fragments in the DSi-poised library are able to make efficient hydrophobic interactions to facilitate binding. **(a):** interactions were calculated using the residue IFP method. **(b):** fragments have been calculated using the atomic IFP method.

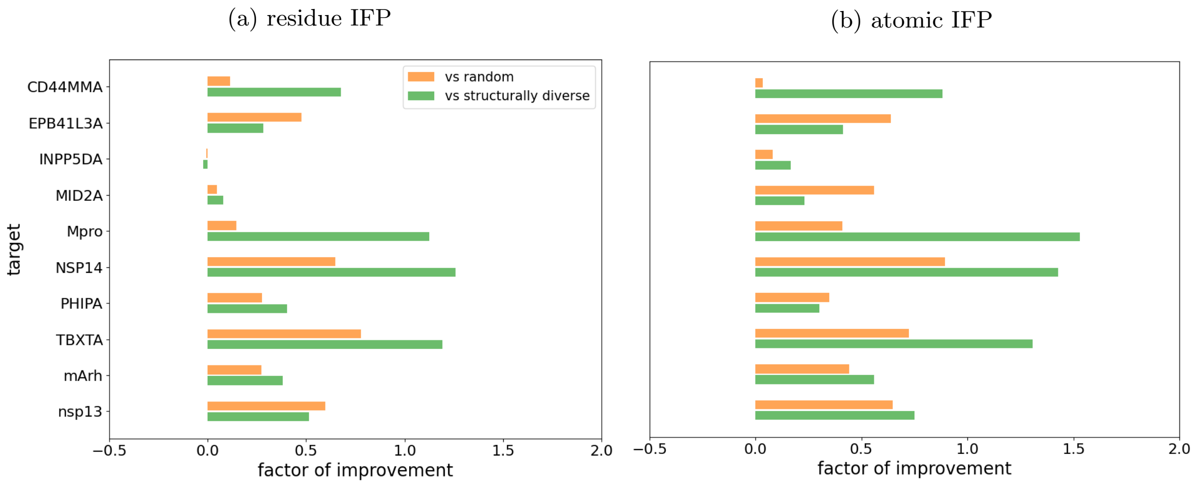

Figure S5: Factor of information improvement when using 100 functionally diverse fragments compared with random and structurally diverse libraries. **(a):** fragments have been ranked using the residue IFP method. **(b):** fragments have been ranked using the atomic IFP method.

2 Molecules included in analysis

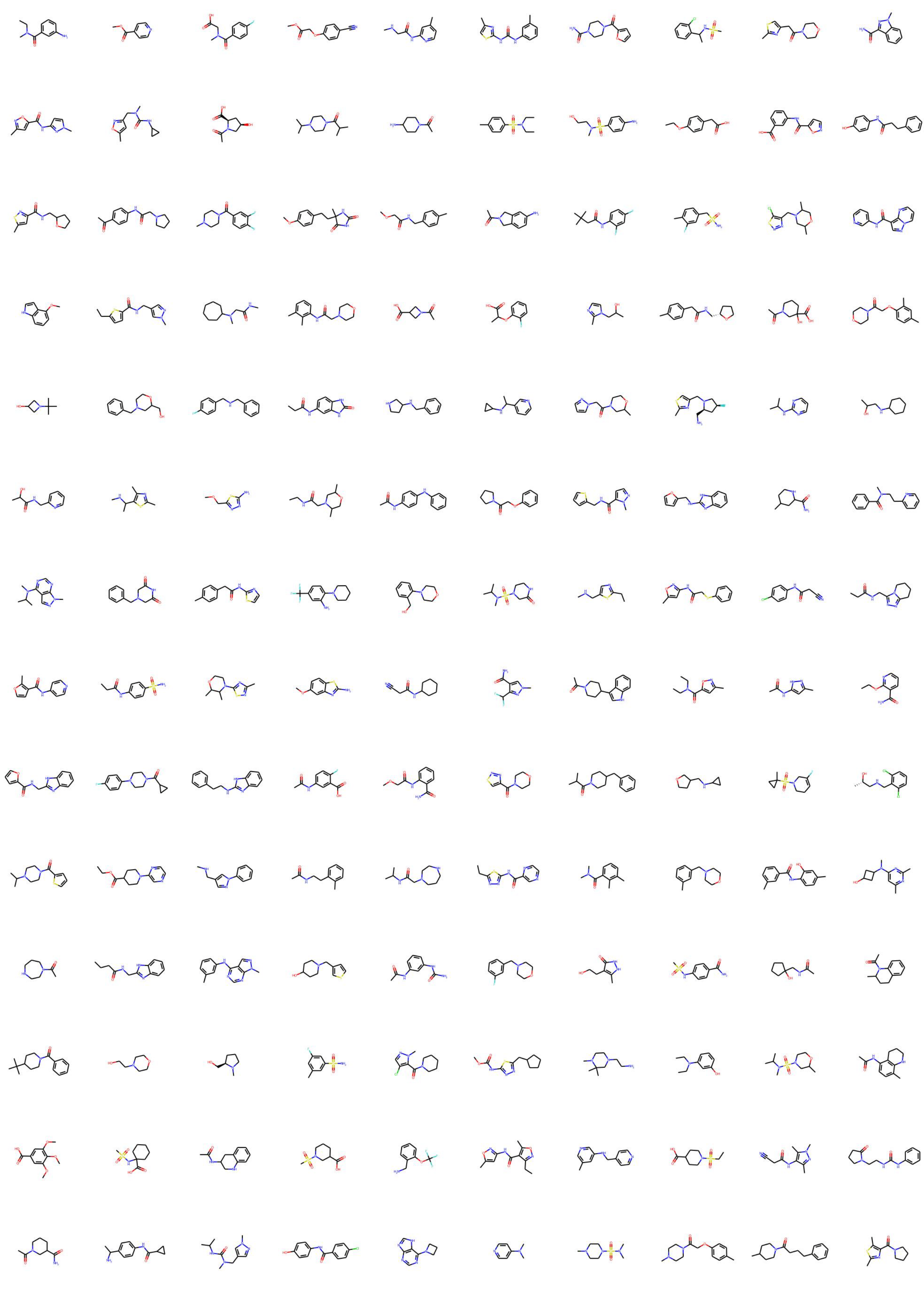

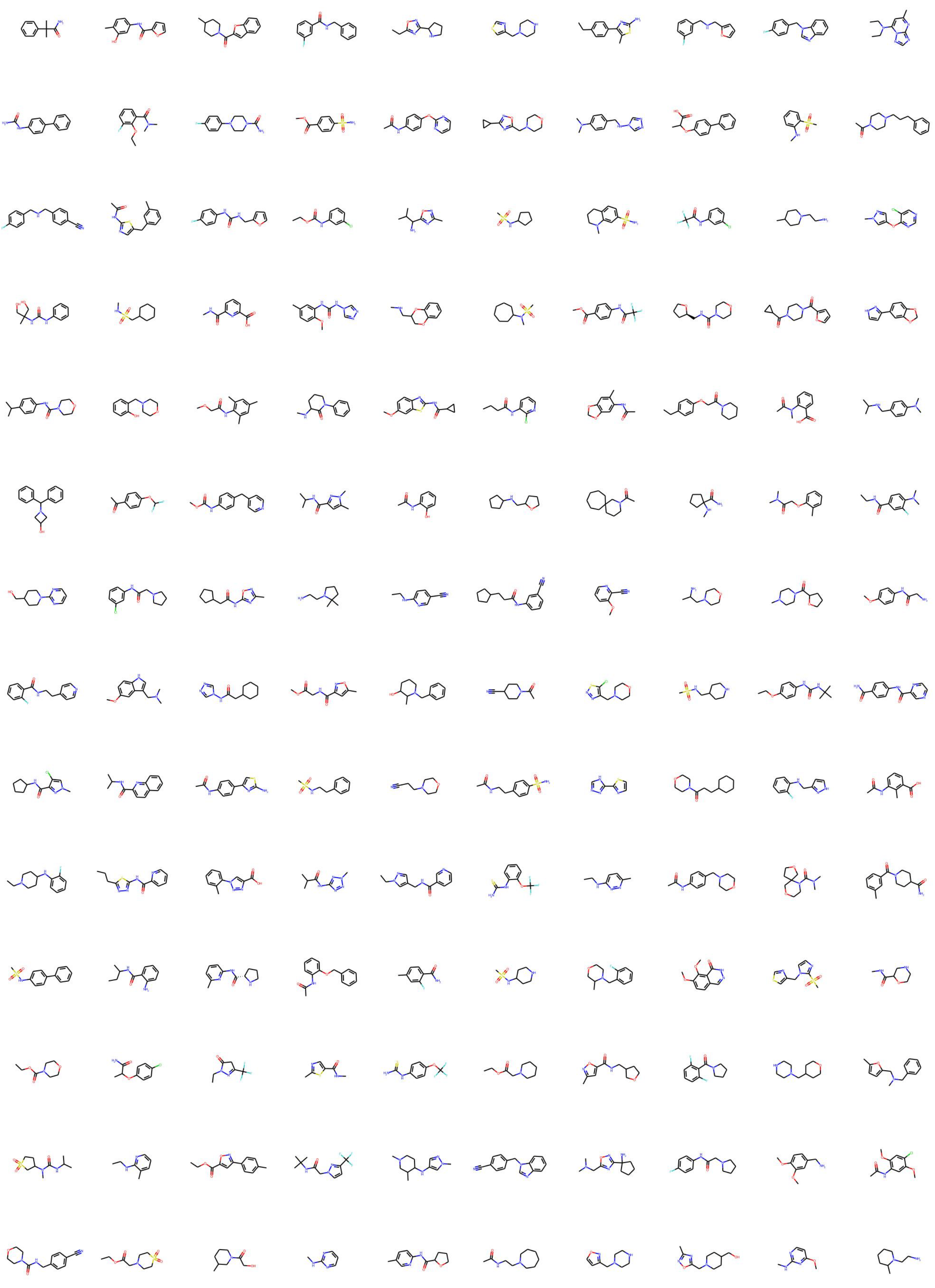

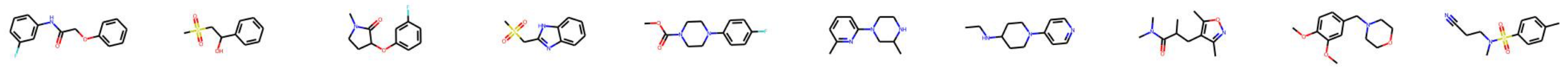
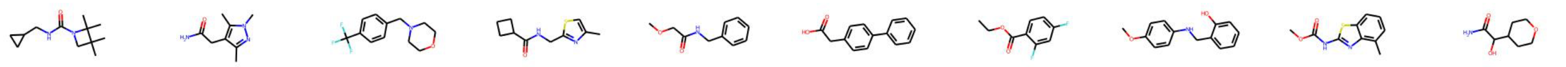
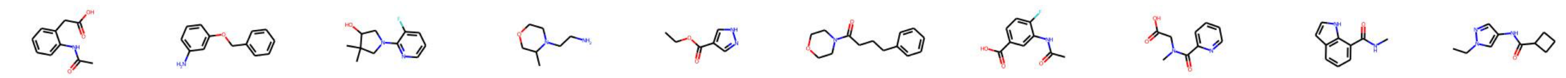
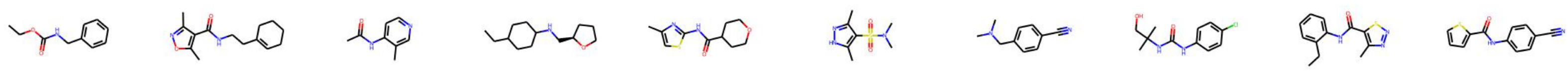
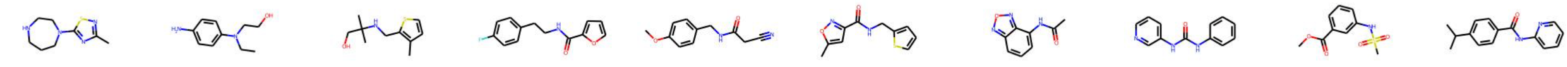
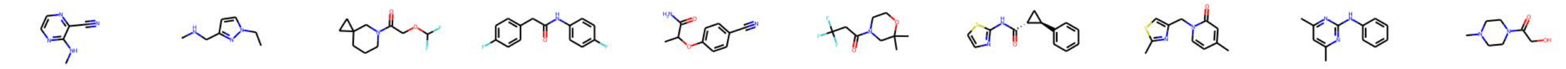
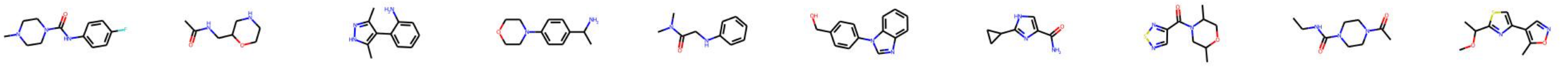
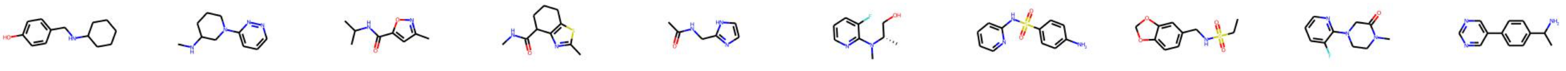
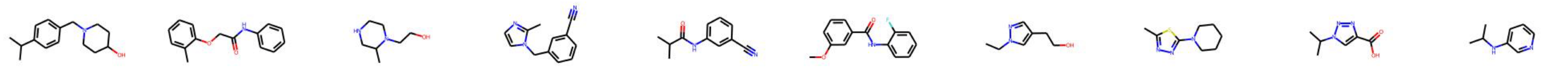
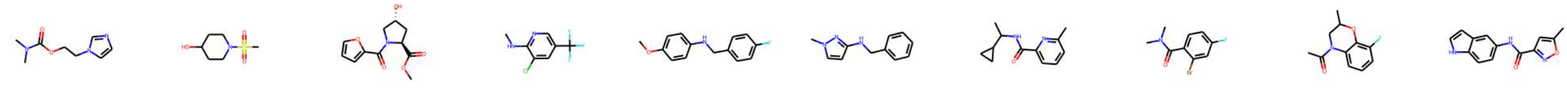
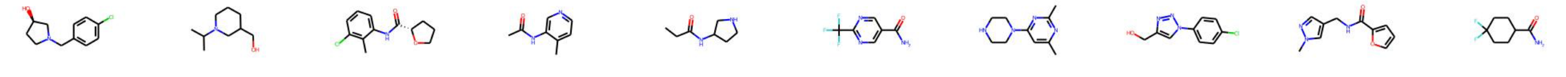
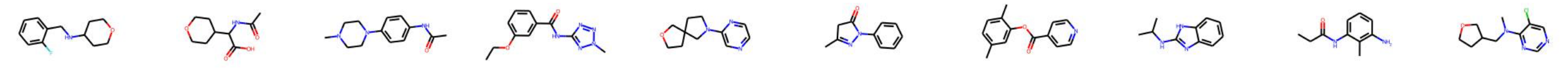
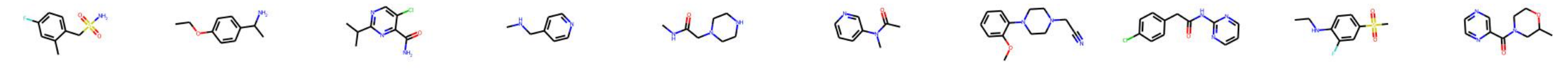
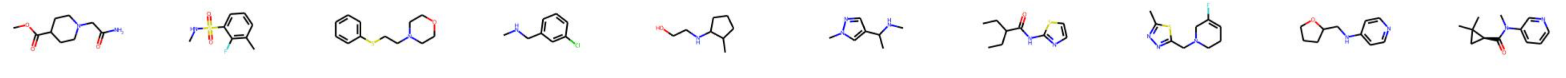

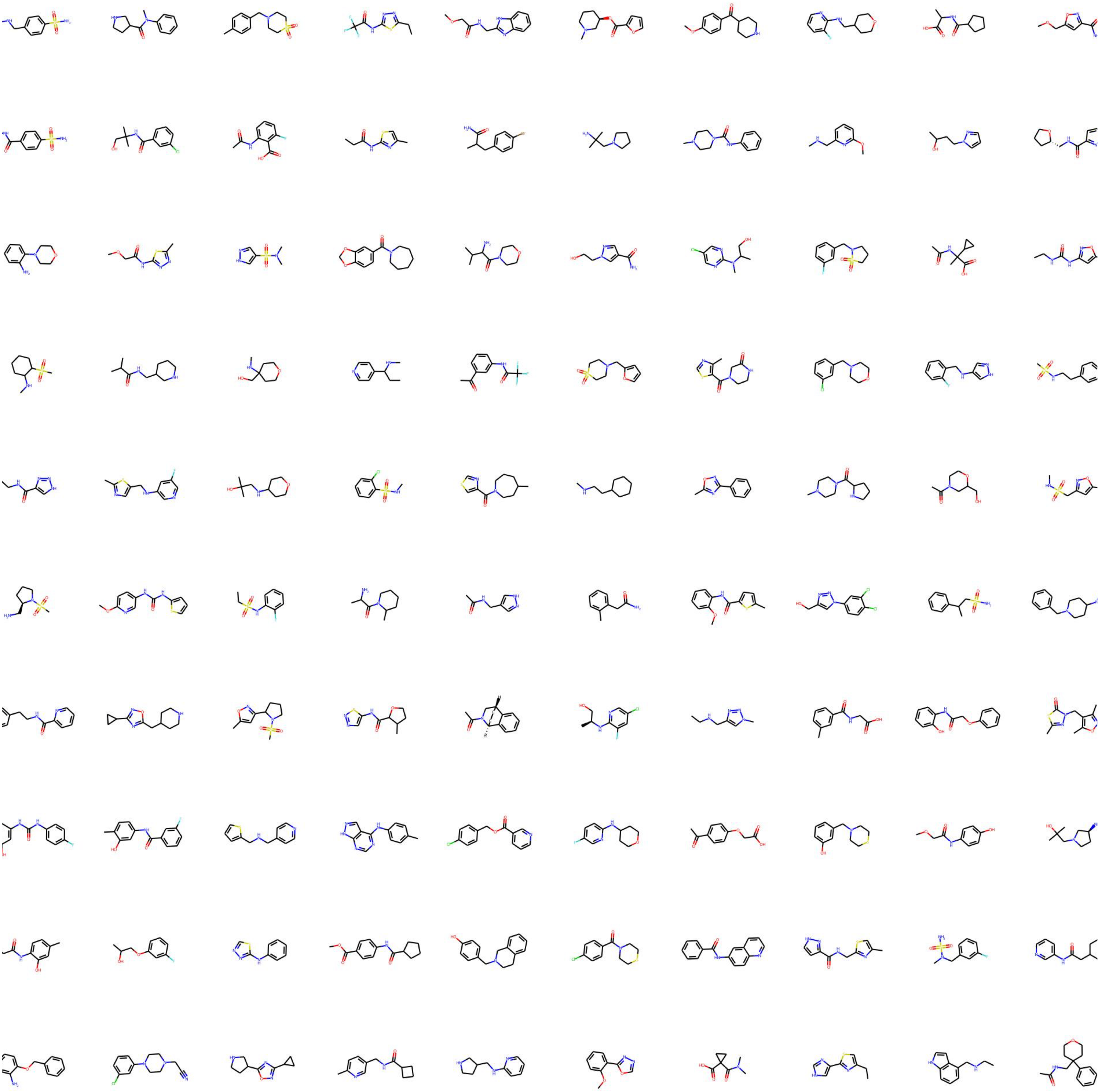

References

[1] XChem at Diamond Light Source. *The Diamond-SGC iNext Poised library.*

[2] Jan Wollenhaupt, Alexander Metz, Tatjana Barthel, Gustavo M A Lima, Andreas Heine, Uwe Mueller, Gerhard Klebe, and Manfred S Weiss. "F2X-Universal and F2X-Entry: structurally diverse compound libraries for crystallographic fragment screening". In: *Structure* (2020). DOI: `https://doi.org/10.1016/j.str.2020.04.019`.

[3] Dávid Bajusz, Warren S Wade, Grzegorz Satała, Andrzej J Bojarski, Janez Ilaš, Jessica Ebner, Florian Grebien, Henrietta Papp, Ferenc Jakab, Alice Douangamath, Daren Fearon, Frank von Delft, Marion Schuller, Ivan Ahel, Amanda Wakefield, Sándor Vajda, János Gerencsér, Péter Pallai, and György M Keserű. "Exploring protein hotspots by optimized fragment pharmacophores". In: *Nature Communications* 12.1 (2021), p. 3201. DOI: `10.1038/s41467-021-23443-y`.

[4] Daniel J Wood, J Daniel Lopez-Fernandez, Leanne E Knight, Islam Al-Khawaldeh, Conghao Gai, Shengying Lin, Mathew P Martin, Duncan C Miller, Céline Cano, Jane A Endicott, Ian R Hardcastle, Martin E M Noble, and Michael J Waring. "FragLites—minimal, halogenated fragments displaying pharmacophore doublets. An efficient approach to druggability assessment and hit generation". In: *Journal of Medicinal Chemistry* 62.7 (2019), pp. 3741–3752. DOI: `10.1021/acs.jmedchem.9b00304`.

[5] Alice Douangamath, Daren Fearon, Paul Gehrtz, Tobias Krojer, Petra Lukacik, C David Owen, Efrat Resnick, Claire Strain-Damerell, Anthony Aimon, Péter Ábrányi-Balogh, José Brandaõ-Neto, Anna Carbery, Gemma Davison, Alexandre Dias, Thomas D Downes, Louise Dunnett, Michael Fairhead, James D Firth, S Paul Jones, Aaron Keely, György M Keserü, Hanna F Klein, Mathew P Martin, Martin E M Noble, Peter O, Ailsa Powell, Rambabu Reddi, Rachael Skyner, Matthew Snee, Michael J Waring, Conor Wild, Nir London, Frank von Delft, and Martin A Walsh. "Crystallographic and electrophilic fragment screening of the SARS-CoV-2 main protease". In: *Nature Communications* 11.5047 (2020). DOI: `https://doi.org/10.1038/s41467-020-18709-w`.

[6] Sarah L Kidd, Elaine Fowler, Till Reinhardt, Thomas Compton, Natalia Mateu, Hector Newman, Dom Bellini, Romain Talon, Joseph McLoughlin, Tobias Krojer, Anthony Aimon, Anthony Bradley, Michael Fairhead, Paul Brear, Laura Díaz-Sáez, Katherine McAuley, Hannah F Sore, Andrew Madin, Daniel H O'Donovan, Kilian V M Huber, Marko Hyvönen, Frank von Delft, Christopher G Dowson, and David R Spring. "Demonstration of the utility of DOS-derived fragment libraries for rapid hit derivatisation in a multidirectional fashion". In: *Chem. Sci.* 11.39 (2020), pp. 10792–10801. DOI: `10.1039/D0SC01232G`.

[7] Thomas D Downes, S Paul Jones, Hanna F Klein, Mary C Wheldon, Masakazu Atobe, Paul S Bond, James D Firth, Ngai S Chan, Laura Waddelove, Roderick E Hubbard, David C Blakemore, Claudia De Fusco, Stephen D Roughley, Lewis R Vidler, Maria Ann Whatton, Alison J.-A. Woolford, Gail L Wrigley, and Peter O'Brien. "Design and synthesis of 56 shape-diverse 3D fragments". In: *Chemistry – A European Journal* 26.41 (2020), pp. 8969–8975. DOI: `https://doi.org/10.1002/chem.202001123`.

[8] Daniel J Foley, Philip G E Craven, Patrick M Collins, Richard G Doveston, Anthony Aimon, Romain Talon, Ian Churcher, Frank vonDelft, Stephen P Marsden, and Adam Nelson. "Synthesis and demonstration of the biological relevance of sp3-rich scaffolds distantly related to natural product frameworks". In: *Chemistry – A European Journal* 23.60 (2017), pp. 15227–15232. DOI: `10.1002/chem.201704169`.

[9] Anthony Aimon, George Karageorgis, Jacob Masters, Mark Dow, Philip G E Craven, Martin Ohsten, Anthony Willaume, Rémy Morgentin, Nicolas Ruiz-Llamas, Hugues Lemoine, Tuomo Kalliokoski, Andrew J Eatherton, Daniel J Foley, Stephen P Marsden, and Adam Nelson. "Realisation of small molecule libraries based on frameworks distantly related to natural products". In: *Org. Biomol. Chem.* 16.17 (2018), pp. 3160–3167. DOI: 10.1039/C8OB00688A.

[10] Marc O'Reilly, Anne Cleasby, Thomas G Davies, Richard J Hall, R Frederick Ludlow, Christopher W Murray, Dominic Tisi, and Harren Jhoti. "Crystallographic screening using ultra-low-molecular-weight ligands to guide drug design." eng. In: *Drug discovery today* 24.5 (2019), pp. 1081–1086. DOI: 10.1016/j.drudis.2019.03.009.

[11] Richard J Hall, Paul N Mortenson, and Christopher W Murray. "Efficient exploration of chemical space by fragment-based screening". eng. In: *Progress in Biophysics and Molecular Biology* 116.2-3 (2014), pp. 82–91. DOI: 10.1016/j.pbiomolbio.2014.09.007.

[12] Aaron Keeley, László Petri, Péter Ábrányi-Balogh, and György M Keserű. "Covalent fragment libraries in drug discovery". In: *Drug Discovery Today* 25.6 (2020), pp. 983–996. DOI: https://doi.org/10.1016/j.drudis.2020.03.016.

[13] A Keeley, P Ábrányi-Balogh, and G M Keserű. "Design and characterization of a heterocyclic electrophilic fragment library for the discovery of cysteine-targeted covalent inhibitors". In: *Med. Chem. Commun.* 10.2 (2019), pp. 263–267. DOI: 10.1039/C8MD00327K.

[14] Efrat Resnick, Anthony Bradley, Jinrui Gan, Alice Douangamath, Tobias Krojer, Ritika Sethi, Paul P Geurink, Anthony Aimon, Gabriel Amitai, Dom Bellini, James Bennett, Michael Fairhead, Oleg Fedorov, Ronen Gabizon, Jin Gan, Jingxu Guo, Alexander Plotnikov, Nava Reznik, Gian Filippo Ruda, Laura Díaz-Sáez, Verena M Straub, Tamas Szommer, Srikannathasan Velupillai, Daniel Zaidman, Yanling Zhang, Alun R Coker, Christopher G Dowson, Haim M Barr, Chu Wang, Kilian V M Huber, Paul E Brennan, Huib Ovaa, Frank von Delft, and Nir London. "Rapid covalent-probe discovery by electrophile-fragment screening". In: *Journal of the American Chemical Society* 141.22 (2019), pp. 8951–8968. DOI: 10.1021/jacs.9b02822.

[15] Peter S Kutchukian, Anne Mai Wassermann, Mika K Lindvall, S Kirk Wright, Johannes Ottl, Jaison Jacob, Clemens Scheufler, Andreas Marzinzik, Natasja Brooijmans, and Meir Glick. "Large scale meta-analysis of fragment-based screening campaigns: privileged fragments and complementary technologies". In: *Journal of Biomolecular Screening* 5 (), pp. 588–596. DOI: 10.1177/1087057114565080.

[16] Nathan Fuller, Loredana Spadola, Scott Cowen, Joe Patel, Heike Schönherr, Qing Cao, Andrew McKenzie, Fredrik Edfeldt, Al Rabow, and Robert Goodnow. "An improved model for fragment-based lead generation at AstraZeneca". In: *Drug Discovery Today* 21.8 (2016), pp. 1272–1283. DOI: https://doi.org/10.1016/j.drudis.2016.04.023.

[17] Chembridge. *Chembridge Fragment Library.* URL: https://www.chembridge.com/screening_libraries/fragment_library/.

[18] Asinex. *Asinex Fragment Library.* URL: https://www.asinex.com/?page_id=97.

[19] Life Chemicals. *Life Chemicals Fragment Libraries.* URL: https://lifechemicals.com/screening-libraries/fragment-libraries.

[20] Prestwick. *Preswick Drug-Fragment Library.* URL: https://www.prestwickchemical.com/screening-libraries/prestwick-drug-fragment-library/.

[21] Selcia. *Selcia Fragment Library.* URL: https://www.cambridgemedchemconsulting.com/DDResources/Hit_iden/frag_coll_profiles_files/SelciaFragmentLibraryPoster_FBLD2010_low\%20res.pdf.

[22] Timetec. *Timetec Fragment Library.* URL: https://www.timtec.net/fbl-fragment-based-library.html.

[23] Zenobia. *Zenobia Discovery-ZEN Fragment Library.* URL: https://www.zenobiafragments.com/product-page/discovery-zen-library-1.

[24] Alan E Bilsland, Kirsten McAulay, Ryan West, Angelo Pugliese, and Justin Bower. "Automated generation of novel fragments using screening data, a dual SMILES autoencoder, transfer learning and syntax correction". In: *Journal of Chemical Information and Modeling* 61.6 (2021), pp. 2547–2559. DOI: 10.1021/acs.jcim.0c01226.

[25] Enamine. *Enamine Fragment Collection.* URL: https://enamine.net/compound-libraries/fragment-libraries/.

[26] Christopher G Parker, Andrea Galmozzi, Yujia Wang, Bruno E Correia, Kenji Sasaki, Christopher M Joslyn, Arthur S Kim, Cullen L Cavallaro, R Michael Lawrence, Stephen R Johnson, Iñigo Narvaiza, Enrique Saez, and Benjamin F Cravatt. "Ligand and target discovery by fragment-based screening in human cells". In: *Cell* 168.3 (2017), 527–541.e29. DOI: 10.1016/j.cell.2016.12.029.

[27] EU openscreen. *EU openscreene Fragment Library.* URL: https://www.eu-openscreen.eu/.

[28] Maybridge. *Maybridge Fragment Library.* URL: https://www.thermofisher.com/uk/en/home/industrial/pharma-biopharma/drug-discovery-development/screening-compounds-libraries-hit-identification/maybridge-fragment-libraries.html.

[29] I-Jen Chen and Roderick E Hubbard. "Lessons for fragment library design: analysis of output from multiple screening campaigns". In: *Journal of Computer-Aided Molecular Design* 23.8 (2009), pp. 603–620. DOI: 10.1007/s10822-009-9280-5.

[30] Wan F Lau, Jane M Withka, David Hepworth, Thomas V Magee, Yuhua J Du, Gregory A Bakken, Michael D Miller, Zachary S Hendsch, Venkataraman Thanabal, Steve A Kolodziej, Li Xing, Qiyue Hu, Lakshmi S Narasimhan, Robert Love, Maura E Charlton, Samantha Hughes, Willem P van Hoorn, and James E Mills. "Design of a multi-purpose fragment screening library using molecular complexity and orthogonal diversity metrics". In: *Journal of Computer-Aided Molecular Design* 25.7 (2011), p. 621. DOI: 10.1007/s10822-011-9434-0.

[31] Maciej Wójcikowski, Piotr Zielenkiewicz, and Pawel Siedlecki. "Open drug discovery toolkit (ODDT): a new open-source player in the drug discovery field". In: *Journal of Cheminformatics* 7.1 (2015), p. 26. DOI: 10.1186/s13321-015-0078-2.

[32] Suneale Banerji, Alan J Wright, Martin Noble, David J Mahoney, Iain D Campbell, Anthony J Day, and David G Jackson. "Structures of the Cd44–hyaluronan complex provide insight into a fundamental carbohydrate-protein interaction". In: *Nature Structural Molecular Biology* 14.3 (2007), pp. 234–239. DOI: 10.1038/nsmb1201.

[33] William Bradshaw, Vittorio Katis, and Opher Gileadi. *EPB41L3; A Target Enabling Package.* 2020. DOI: 10.5281/ZENODO.4429638.

[34] William Bradshaw, Richard Priestley, Juliane Obst, Hazel Hall-Roberts, Anna Cederbalk, Paul Brennan, Emma Mead, Elena Di Daniel, John Davis, and Opher Gileadi. *SH2-containing-inositol-5-phosphatases (INPP5D); A Target Enabling Package.* 2020. DOI: 10.5281/ZENODO.4429262.

[35] Uniprot. *MID2 - Uniprot.*

[36] The UniProt Consortium. “UniProt: the universal protein knowledgebase in 2021”. In: *Nucleic Acids Research* 49.D1 (2020), pp. D480–D489. DOI: 10.1093/nar/gkaa1100.

[37] Natacha S Ogando, Jessika C Zevenhoven-Dobbe, Yvonne van der Meer, Peter J Bredenbeek, Clara C Posthuma, Eric J Snijder, and Tom Gallagher. “The enzymatic activity of the nsp14 exoribonuclease is critical for replication of MERS-CoV and SARS-CoV-2”. In: *Journal of Virology* 94.23 (2020), e01246–20. DOI: 10.1128/JVI.01246-20.

[38] Oakley B. Cox, Tobias Krojer, Patrick Collins, Octovia Monteiro, Romain Talon, Anthony Bradley, Oleg Fedorov, Jahangir Amin, Brian D. Marsden, John Spencer, and et al. *Human Pleckstrin Homology domain Interacting Protein (PHIP); A Target Enabling Package.* 2016. DOI: 10.5281/ZENODO.4134700.

[39] Joseph Newman, Angeline Gavard, Hazel Aitkenhead, David Drewry, Charles Lin, and Opher Gileadi. *Human T-box transcription factor T (Brachyury); A Target Enabling Package.* 2020. DOI: 10.5281/ZENODO.4265383.

[40] Marion Schuller, Galen J. Correy, Stefan Gahbauer, Daren Fearon, Taiasean Wu, Roberto Efraín Díaz, Iris D. Young, Luan Carvalho Martins, Dominique H. Smith, Ursula Schulze-Gahmen, Tristan W. Owens, Ishan Deshpande, Gregory E. Merz, Aye C. Thwin, Justin T. Biel, Jessica K. Peters, Michelle Moritz, Nadia Herrera, Huong T. Kratochvil, null null, Anthony Aimon, James M. Bennett, Jose Brandao Neto, Aina E. Cohen, Alexandre Dias, Alice Douangamath, Louise Dunnett, Oleg Fedorov, Matteo P. Ferla, Martin R. Fuchs, Tyler J. Gorrie-Stone, James M. Holton, Michael G. Johnson, Tobias Krojer, George Meigs, Ailsa J. Powell, Johannes Gregor Matthias Rack, Victor L. Rangel, Silvia Russi, Rachael E. Skyner, Clyde A. Smith, Alexei S. Soares, Jennifer L. Wierman, Kang Zhu, Peter O’Brien, Natalia Jura, Alan Ashworth, John J. Irwin, Michael C. Thompson, Jason E. Gestwicki, Frank von Delft, Brian K. Shoichet, James S. Fraser, and Ivan Ahel. “Fragment binding to the Nsp3 macrodomain of SARS-CoV-2 identified through crystallographic screening and computational docking”. In: *Science Advances* 7.16 (2021), eabf8711. DOI: 10.1126/sciadv.abf8711.

[41] Joseph Newman, Yuliana Yosaatmadja, Alice Douangamath, Frank Von Delft, and Opher Gileadi. *SARS-CoV-2 NSP13; A Target Enabling Package.* 2020. DOI: 10.5281/ZENODO.4449959.

[42] Ian Sillitoe, Nicola Bordin, Natalie Dawson, Vaishali P Waman, Paul Ashford, Harry M Scholes, Camilla S M Pang, Laurel Woodridge, Clemens Rauer, Neeladri Sen, Mahnaz Abbasian, Sean Le Cornu, Su Datt Lam, Karel Berka, Ivana Hutařová Varekova, Radka Svobodova, Jon Lees, and Christine A Orengo. “CATH: increased structural coverage of functional space”. In: *Nucleic Acids Research* 49.D1 (Nov. 2020), pp. D266–D273. DOI: 10.1093/nar/gkaa1079. eprint: https://academic.oup.com/nar/article-pdf/49/D1/D266/35364652/gkaa1079.pdf.

[43] Greg Landrum. *RDKit: Open-source cheminformatics.*

[44] Renato Ferreira de Freitas and Matthieu Schapira. “A systematic analysis of atomic protein-ligand interactions in the PDB.” eng. In: *MedChemComm* 8.10 (2017), pp. 1970–1981. DOI: 10.1039/c7md00381a.

[45] Fabrizio Giordanetto, Chentian Jin, Lindsay Willmore, Miklos Feher, and David E Shaw. “Fragment hits: what do they look like and low do they bind?” eng. In: *Journal of Medicinal Chemistry* 62.7 (2019), pp. 3381–3394. DOI: 10.1021/acs.jmedchem.8b01855.
